# Impact of Acid Dopants (HCl vs. H_2_SO_4_) on the Structure, Performance, and Reusability of Bio‐Based Luffa/Polyaniline Composites for Methylene Blue Adsorption

**DOI:** 10.1002/open.70185

**Published:** 2026-05-21

**Authors:** Rayane Mehennaoui, Soraya Merzouki, Salah Neghmouche Nacer, Abderrahmane Mehennaoui, Stefania Garzoli, Fadila Louafi

**Affiliations:** ^1^ Research Unit of Environmental and Molecular Structural Chemistry (CHEMS) University of Constantine 1 Constantine Algeria; ^2^ University Center Mila Mila Algeria; ^3^ Department of Chemistry Faculty of Exact Sciences University of El Oued El Oued Algeria; ^4^ University of Constantine 3 Salah Boubnider Constantine Algeria; ^5^ Department of Chemistry and Technologies of Drug Sapienza University Rome Italy

**Keywords:** acid doping, adsorption, *Luffa cylindrica*/polyaniline composites, methylene blue, sustainable materials, wastewater treatment

## Abstract

Sustainable and low‐cost Luffa cylindrica/polyaniline (LC/PAN) composite adsorbents were developed for the removal of methylene blue (MB) from aqueous solutions. The effect of acid dopants (hydrochloric acid and sulfuric acid) on the structural properties and adsorption performance of the composites was systematically investigated. LC/PAN materials were synthesized via a dual‐acid doping approach and characterized using FT‐IR, Raman, SEM, XRD, TGA, and BET techniques. Batch adsorption experiments were conducted to examine the influence of adsorbent dosage, initial dye concentration, pH, and temperature, while adsorption behavior was analyzed using kinetic, isotherm, and thermodynamic models. Although the HCl‐doped LC/PAN exhibited a higher BET surface area (20.8 m^2^/g), the H_2_SO_4_‐doped composite showed superior MB adsorption capacity (*Q*_max _ ≈  10 mg/g), indicating that surface chemistry plays a more critical role than total porosity. The adsorption process was rapid, followed pseudo‐second‐order kinetics and the Langmuir isotherm, and was spontaneous and exothermic. These results demonstrate that acid dopant selection is a key factor in tailoring LC/PAN biocomposites for efficient and sustainable dye adsorption.

## Graphical abstract

Evaluation of the impact of acidic dopants (HCl vs. H_2_SO_4_) on bio‐derived luffa/polyaniline composites for methylene blue adsorption.

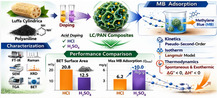

## 1 Introduction

Water is the foundation of life on Earth. It is indispensable to ecosystems, supporting biodiversity, regulating natural cycles, and sustaining human societies. Beyond its ecological role, water is essential to the global economy, underpinning agriculture, industry, energy production, and commerce.

However, rapid population growth, urbanization, and industrial development have placed immense pressure on water resources. Industrial activities alone discharge an estimated 300–500 million tonnes of pollutants into water annually (UNESCO) [1]. Among these pollutants, synthetic dyes are of particular concern. Used extensively in textiles, food, cosmetics, and pharmaceuticals [2], they are often released into waterways untreated or only partially treated [3]. Their intense coloration reduces sunlight penetration, diminishing dissolved oxygen levels and threatening aquatic ecosystems [4]. Many dyes also exhibit high toxicity and carcinogenicity, posing risks to both environmental and human health [5]. Over the past decade, improving water quality and safeguarding aquatic resources have therefore become major global challenges.

Various methods have been employed to treat dye‐contaminated wastewater, including biological approaches (using enzymes or microorganisms) [6], physicochemical methods (notably adsorption) [7], and chemical techniques (such as advanced oxidation processes) [6, 7]. Recently, electro‐adsorption has emerged as a promising technology for desalination and removal of dyes [8]. In addition, machine learning has been applied to simulate and predict the adsorption performance of activated carbons for removing methylene blue from wastewater, providing an innovative and efficient approach for optimizing treatment processes.

Yet, these conventional processes often face limitations such as incomplete pollutant removal, high energy or reagent demands, and production of toxic sludge [9]. Consequently, research has increasingly focused on cost‐effective and sustainable alternatives. Adsorption, in particular, has emerged as a leading option due to its simplicity, efficiency, and adaptability [10].

To enhance sustainability, biosorbents derived from agricultural byproducts and natural materials have gained attention. These materials combine environmental friendliness with low cost and low carbon footprint, making them attractive for large‐scale wastewater treatment [11]. Reported examples include cotton waste, rice husk [12], palm fruit bundle [13], jackfruit [14], castor seed husk [15], wood [16], orange peel [17], mansonia sawdust [18], and loofah [19].

*Luffa cylindrica* (LC) represents a particularly promising biosorbent. This abundantly available climbing plant, widespread in tropical, subtropical, and Mediterranean regions, is rich in lignocellulosic fibers. Its naturally porous structure [11], high stability, and fast sorption kinetics [20] make it suitable for adsorption applications. Furthermore, the hydroxyl groups present in its fibers enable chemical modifications that can enhance adsorption capacity. To further boost its performance, recent research has explored functionalization with conductive polymers such as polyaniline (PAN), producing composites with tailored properties and improved dye removal efficiency.

Conductive polymers have attracted considerable attention due to their unique chemical and physical properties, low cost, and wide range of applications. Among them, PAN stands out for its multifunctionality, high chemical stability [21], adjustable porosity, and excellent redox reversibility. A critical step in its synthesis is doping with protonic acids, which convert it into its conductive emeraldine salt form. The dopant choice strongly affects the polymer's morphology, crystallinity, and surface chemistry, thereby influencing adsorption behavior.

Previous studies [22] have confirmed the feasibility of combining LC with PAN to form composites for heavy metal removal. Yet, the influence of specific acid dopants on the structure, surface properties, and adsorption mechanisms of LC/PAN composites remains poorly understood. Addressing this knowledge gap is essential to guide the rational design of bio‐based adsorbents with optimized performance.

In this work, we report the synthesis and characterization of LC/PAN composites doped with two acids, hydrochloric acid (HCl) and sulfuric acid (H_2_SO_4_). Advanced techniques (FT‐IR, Raman, SEM, XRD, TGA, BET) are used to investigate how doping modifies the composites’ structure and surface properties. We further assess their adsorption efficiency toward methylene blue (MB), a model cationic dye, through kinetic, isotherm, and thermodynamic studies. By establishing clear correlations between material structure and adsorption performance, this study provides new insights into the design of sustainable, low‐cost sorbents, contributing directly to UN Sustainable Development Goals, particularly Clean Water and Sanitation and Responsible Consumption and Production.

## 2 Materials and Methods

### 2.1 Materials

In this work, we used analytical grade reagents, Aniline C_6_H_5_NH_2_ (was doubly distilled), ammonium persulfate (NH_4_)_2_S_2_O_8_, hydrochloric acid (HCl), sulfuric acid (H_2_SO_4_), and sodium hydroxide (NaOH) were purchased from Biochrom Chemopharma and used without further purification. Ethanol Absolute 99.8% were purchased from VWR Analar Normapur. The dye methylene blue (MB) was obtained from Fluka (96%), and the Luffa cylindrica was bought from a specialty store in Constantine and made locally.

### 2.2 Measuring Instruments

The measuring instruments used in this study are:

• The pH‐meter “HANNA pH211.”
• The UV–Visible spectrophotometer “VWR UV‐1600PC.”

### 2.3 Preparation of Methylene Blue (MB) Solution

Cationic dye stock solutions: Methylene Blue (MB) is prepared by dissolving 100 mg of (MB) in 1 L of distilled water, and the dye is highly soluble in water. Then, through dilution, the solution was prepared with the required initial concentrations of dye from 2 to 28 ppm. The pH of the solution was adjusted by adding HCl (0.1 M) or NaOH (0.1 M).

### 2.4 Preparation of Adsorbents Based on Luffa cylindrica/Polyaniline

Drawing inspiration from the polyaniline preparation methods reported by Ravi et al. [23], we established our methodology for coating *Luffa cylindrica*, which was carried out as follows:

To get rid of unwanted particles, sand and dust that may be carried by the *L*uffa cylindrica. The Luffa C was washed several times with tap water until the water is clear and free of impurities, after which it was rinsed with distilled water and then dried in the oven at 120°C for 30 min.

Then, we put 1g of Luffa in a beaker that was contain 150 mL of HCl (1M) under stirring constant for 30 min after that we put 10.35 mL of Aniline (0.7 M), The solution was kept under stirring for 30 min. We added 4.5 g of ammonium persulfate (0.7 M) to the other flask that held 50 mL of HCl (1 M). Finally, we placed the final beaker inside the luffa‐containing beaker, the molar ratio of aniline and APS (acting as an oxidizing agent) was [1:1]. Immediately, parafilm and aluminum paper were placed over the beaker.

**FIGURE 1:**
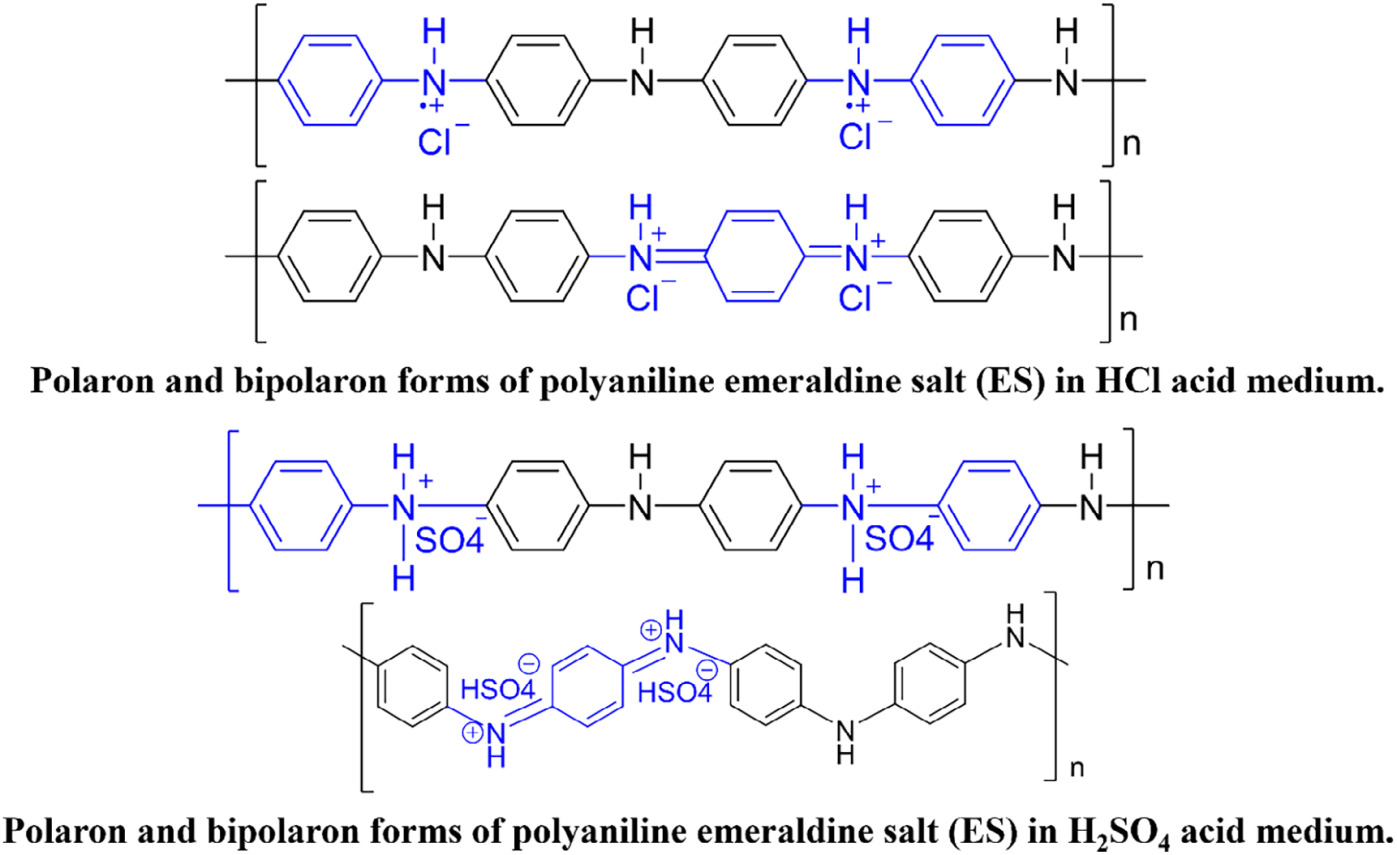
Polyaniline (PAN) doping in protonic acid medium (PAN emeraldine salt).

For a full day, the solution was left at room temperature in the dark while being constantly stirred at 500 rpm. The final composite material (LC/PAN) was filtered, rinsed with distilled water, dried in an oven set to 120°C for 30 min until constant weight, and then kept for later use. Same protocol was repeated for H_2_SO_4_ (1M) (Figure 1) [24].

### 2.5 Characterization of LC/PAN Composites

The structural, morphological, and thermal properties of the prepared LC/PAN composites were investigated using various analytical techniques. For solid‐state analyses, the sample was first ground into a fine powder using an agate mortar and pestle. To ensure particle size uniformity, the resulting powder was sieved through a 0.4 mm mesh.

FT‐IR spectra of the samples were recorded in the attenuated total reflection (ATR) mode using a Bruker INVENIO‐R spectrometer equipped with a diamond crystal accessory. The measurements were carried out in the range of 6000–80 cm^−1^ at a resolution of 4 cm^−1^, averaging 30 scans per sample. A DLaTGS detector was used, and the spectra were processed using OPUS software (version 8.2). Samples were analyzed in solid form without any prior treatment by direct contact with the ATR crystal under ambient conditions.

Raman spectroscopy was performed using a DXR Raman Microscope (Thermo Scientific, USA) equipped with a 780 nm laser. The instrument settings included a 400  lines/mm grating, a 50 µm slit aperture, and a laser power of 10.0 mW. The spectral resolution was 1.9285 cm^−1^, and the scanning range was from 50.4675 to 3411.7849 cm^−1^. A fifth‐order polynomial was applied for baseline correction. The exposure time and number of accumulations were adjusted individually for each sample to optimize the spectral quality. No specific sample preparation was required prior to the measurements.

The surface morphology of the samples was characterized using a Scanning Electron Microscope (FEG‐SEM; Apreo 2C, Thermo Fisher Scientific). The analysis was conducted with an accelerating voltage of (5 kV, 10 kV) a spot size of (5, 8), and a working distance of 8.8312 mm. Images were acquired using an Everhart–Thornley Detector (ETD) for secondary electrons (SE) at a magnification of 35,000×. Prior to analysis, the non‐conductive samples were sputter‐coated with a thin layer of copper to prevent surface charging and ensure high‐resolution imaging.

The specific surface area was determined by nitrogen (N_2_) adsorption–desorption analysis at 77 K using a Micromeritics ASAP 2020 Plus instrument. Prior to the measurement, the sample was degassed under vacuum at 150°C for 6 h. The surface area was calculated from the resulting isotherm using the Brunauer–Emmett–Teller (BET) method.

X‐ray diffraction (XRD) analysis was performed on a Rigaku instrument equipped with a Cu Kα radiation source (*λ * =  1.5406 Å), operating at 20 kV and 200  mA. The diffractograms were obtained over a 2*θ* scan range of 10°–90° at a scan speed of 10 s per step with a 0.03° step size. The raw data were plotted using OriginPro 8 software for qualitative analysis. The observed changes in peak positions, intensities, and shapes in the diffractograms after the adsorption process were compared with the initial state of the material and interpreted based on previously published literature.

The thermal stability of the samples was investigated by thermogravimetric analysis (TGA) using a Mettler Toledo TGA/DSC instrument. Samples were heated from room temperature to 900°C at a constant heating rate of 10°C/min under a flowing nitrogen atmosphere.

The pH zero‐point charge (pHzpc) of the LC/PAN in different acids was determined by the method of Aichour et al. [25] with ratio (1:1). 10 mg of LC/PAN was added to 10 mL of distilled water. By adding either HCl (0.1 M) or NaOH (0.1 M), the solution's initial pH (pH_i_) values were approximately changed from 2 to 12. The final pH (pH_f_) of the solution was then recorded after it had been magnetically agitated for 24 h (500 rpm) at room temperature. It was determined using a graphic (pHzpc). The difference between the starting pH (pH_i_) and ending pH (pH_f_) values (ΔpH) might be seen by plotting against pHzpc. The intersection of the resulting curve at ΔpH = 0 is where the pHzpc was found.

### 2.6 Batch Equilibrium Studies

LC/PAN was used as the adsorbent in MB adsorption investigations. A series of 100 mL beakers with a predetermined amount of adsorbent (100 mg) and volume (50 mL in each beaker) of dye solution at a fixed beginning concentration (10 ppm) and a constant solution pH were used for the batch studies. The beaker was then set at room temperature and spinning at 500 rpm on a magnetic stirrer. The samples were examined at specific time intervals. The initial and equilibrium concentrations were measured with a VWR UV‐1600 PC spectrophotometer at wavelengths of 650 nm for MB.

The removal efficiency (*R*%) of the MB process (Equation (1)) and the amount of adsorbent adsorbed at equilibrium *Q*_e_ (mg/g) were determined by (Equation (2)), respectively:

(1) $$R\%=\frac{C_{i}-C_{e}}{C_{i}}\times 100$$

(2) $$Q_{e}=\frac{C_{i}-C_{e}}{W}\times V$$

where *W* is the mass of adsorbent expressed in (g), *V* is the volume of the solution in (L), *C*_i_ is the initial concentrations, and *C*_e_ is the equilibrium concentrations, respectively, expressed in (mg/L).

The experiments were carried out under the effects of several operational parameters, such as the contact time (1–180 min), dose of adsorbent (50–600 mg), initial concentration (2–28 mg/L), pH of the solutions (2–12), and temperature (25°C–55°C), were investigated.

All adsorption experiments were conducted in triplicate under identical experimental conditions to ensure reproducibility. The reported values correspond to the mean of three independent measurements, and the associated error bars represent the standard deviation.

### 2.7 Kinetic and Equilibrium Models

In this study, the adsorption kinetics of MB on LC/PAN were modeled using Three kinetics models: the pseudo‐first‐order, pseudo‐second‐order, intraparticle diffusion. Also, an adsorption process can be expressed using a number of equations to express the equilibrium removal of the adsorption process. This study assessed the dye removal from the aqueous solution using the Langmuir and Freundlich isotherm functions.

### 2.8 Desorption and Regeneration Experiment

In order to create a cost‐effective adsorbent for the removal of MB dye, desorption and regeneration studies were conducted recently [26]. Luffa (100 mg) loaded with MB (10 mg/L) was thoroughly washed with distilled water to remove any non‐adsorbed dye and then dried in the oven at 120°C for 30 min. The biomass was then placed in a beaker containing 50 mL of ethanol. The sample was stirred for 180 min, filtered, washed again with distilled water, and dried in the oven at 120°C for 30 min. The regenerated adsorbent sample was reused in the next cycle of the adsorption experiment, and regenerated experiments were conducted for eight cycles.

## 3 Results and Discussion

### 3.1 Characterization of Adsorbents

**FIGURE 2:**
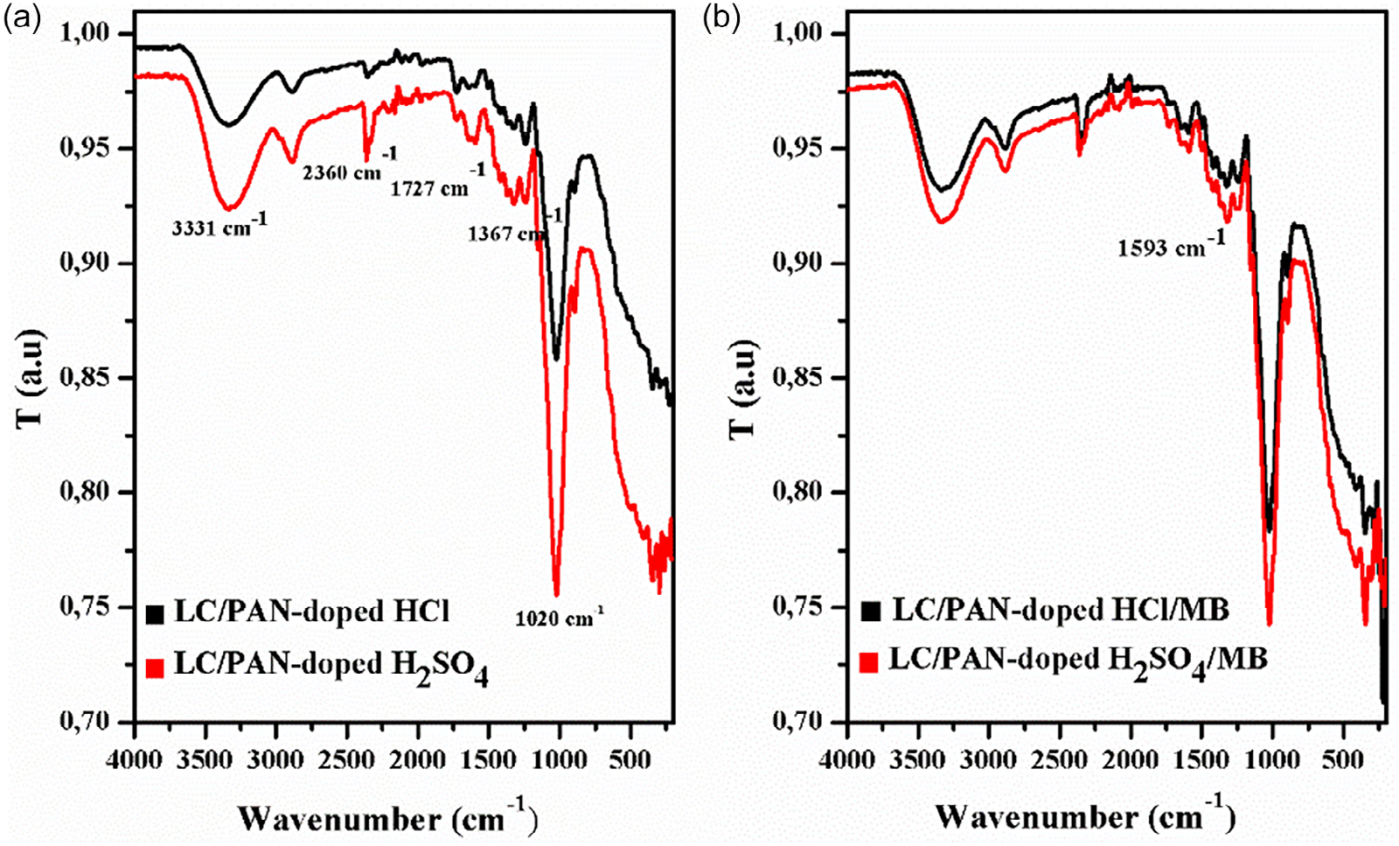
FT‐IR LC/PAN‐doped HCl and H_2_SO_4_. (a) Before adsorption. (b) After adsorption.

**FIGURE 3:**
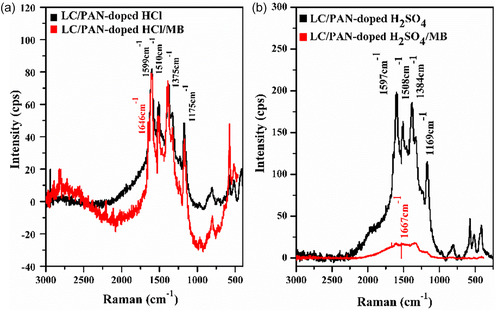
RAMAN (a) LC/PAN‐doped HCl and LC/PAN‐doped HCl/MB and (b) LC/PAN‐doped H_2_SO_4_ and LC/PAN‐doped H_2_SO_4_/MB.

The low standard deviation values obtained indicate good reproducibility and reliability of the adsorption measurements. The chemical structures of LC/PAN‐doped HCl and LC/PAN‐doped H_2_SO_4_ before and after MB adsorption were characterized by FT‐IR and Raman spectroscopies, as reported in Figures 2 and 3.

Figure 2a shows the FT‐IR spectra of both LC/PAN‐doped HCl and LC/PAN‐doped H_2_SO_4_ before MB adsorption. The appearance of a large band with medium intensity at 3331 cm^−1^ suggests an interaction between the amine groups in PAN and the —OH groups in Luffa cylindrica [27]. The band at 2885–2883 cm^−1^ is assigned to the C—H stretching vibration of the phenyl rings [28, 29]. In addition, the peak at 1727 cm^−1^ is linked to the presence of C=O vibrations, whereas the bands in the 2360–1900 cm^−1^ range are attributed to C—H vibrations in the polymer structure [2].

The characteristic bands of PAN are shown at 1640 and 1504 cm^−1^ for LC/PAN‐doped HCl and 1595 and 1499 cm^−1^ for LC/PAN‐doped H_2_SO_4_; are attributed to the C=C stretching pattern of the quinoid and benzenoid ring, respectively [29]; and indicate the presence of the polyaniline in emeraldine form. The C=N and C—N stretching vibrations with aromatic conjugation (benzene and quinine rings) are responsible for the absorption bands at 1367–1240 cm^−1^ [30], whereas the band of emeraldine salt is observed at 1153 cm^−1^ which is thought to represent the typical peak of polyaniline in conductive situations [31]. Besides, the vibrational mode of polaron (—C—NH+•) or bipolaron (—C=NH+•) structures that were created during the doping process is represented by the band at 1020 cm^−1^, and the broad peak is referred to as the “electronic‐like band” [32]. The out‐of‐plane bending of the para‐substituted benzene ring's C—H bond is responsible for the vibration band at 895 cm^−1^ [33, 34], while the vibration peaks at 516–490 cm^−1^ are caused by C—H bond bending vibrations in the 1,4 di‐substituted benzene ring [32].

After MB adsorption, Figure 2b, a number of bands in these spectra experienced positional changes; besides, one of these peaks disappearing (1367 cm^−1^) in LC/PAN‐doped H_2_SO_4_, indicating MB interaction with or near polaronic sites (C—N^+^—), influenced by the counterion SO_4_^2^^−^/HSO_4_^−^, and the new peak appearing in LC/PAN‐doped HCl (1593 cm^−1^), is mainly attributed to the presence of the adsorbed MB molecules themselves, since methylene blue has intense absorption peaks in this zone (~1600 cm^−1^) due to the elongation vibrations of the aromatic C=C and potentially C=N bonds in its structure [35]. This confirms that MB was adsorbed on the adsorbents’ surface, as seen in Figure 2b. These spectroscopic changes are consistent with findings from other studies investigating dye adsorption on polymer composites, where shifts and intensity changes in characteristic bands confirm the formation of adsorbent‐adsorbate complexes through various mechanisms, including electrostatic interactions and hydrogen bonding [35]. The varying spectral responses between (HCl) and (H_2_SO_4_) doped composites further highlight how the specific structural features and surface chemistry, influenced by the dopant, impact their interaction with the dye.

From the Raman resonance spectra of LC/PAN‐doped HCl and LC/PAN‐doped H_2_SO_4_, as shown in Figure 3, it can be seen that before adsorption of the MB contaminant on the two adsorbents, the spectrum shows the characteristic peaks of polyaniline at 1599, 1510, 1375, and 1175 cm^−1^ for LC/PAN‐doped HCl and 1597, 1508, 1384, and 1169 cm^−1^ for LC/PAN‐doped H_2_SO_4_. Peaks at 1599–1597 and 1510–1508 cm^−1^ confirm the presence of polyaniline on the LC surface. Crucially, the distinct shifts and intensity variations observed between LC/PAN‐doped HCl and LC/PAN‐doped H_2_SO_4_ in these characteristic Raman bands, particularly within the quinoid/benzenoid ring regions and polaron bands, are indicative of differential protonation states and variations in the electronic and structural conformation of PAN induced by the respective dopant acids. For instance, while the absorption intensity of LC/PAN‐doped HCl at 1375 cm^−1^ is lower than that of LC/PAN‐doped H_2_SO_4_ at 1384 cm^−1^, this result directly suggests that HCl may not be as effective as H_2_SO_4_ in inducing a high polaron concentration, leading to a potentially less extended conjugated system in the HCl‐doped variant. This fundamental structural difference imparted by the dopant acid is vital for understanding their subsequent adsorption behavior.

After the adsorption of MB, we observed that it influenced the PAN structure and its vibrational modes. This caused shifts in the positions of PAN peaks, accompanied by changes in intensity; differences in the Raman bands of methylene blue (MB) adsorption on LC/PAN‐doped with HCl versus H_2_SO_4_ indicate structural changes and interactions between the dye molecule and the polymer matrix. These observed changes are not merely due to MB presence, but rather reflect how the specific structural and electronic modifications induced by each dopant acid dictate the nature and strength of interaction with the cationic MB molecule. Doping with HCl leads to protonation of the polymer, which affects the MB binding sites and changes the Raman bands of both the dye and the polymer. Doping with H_2_SO_4_ also affects adsorption but causes different protonation/ionization effects; sulfate ions can affect the electronic structure of the system and Raman spectra. H_2_SO_4_ may cause more significant surface chemical changes than the monoprotic HCl, as sulfate ions are likely to change the LC/PAN surface charge and MB adsorption capacity. While both acid dopants enhance MB adsorption, their specific effects differ due to differences in pH and chemical nature. Moreover, the emergence of a new peak at 1646 cm^−1^ in LC/PAN‐doped HCl corroborates the findings in the FT‐IR spectrum, indicating the presence of the adsorbed MB molecules. These distinct vibrational changes, particularly the shifts and intensity alterations in both FT‐IR and Raman, serve as strong spectroscopic evidence for the successful uptake and interaction of MB dye molecules with the LC/PAN composite surface, consistent with various studies on dye‐adsorbent interactions [35, 36]. The differences observed between (HCl) and (H_2_SO_4_) doped composites highlight the subtle but significant impact of the dopant on the polymer's structural conformation and its binding affinity toward the cationic dye.

**FIGURE 4:**
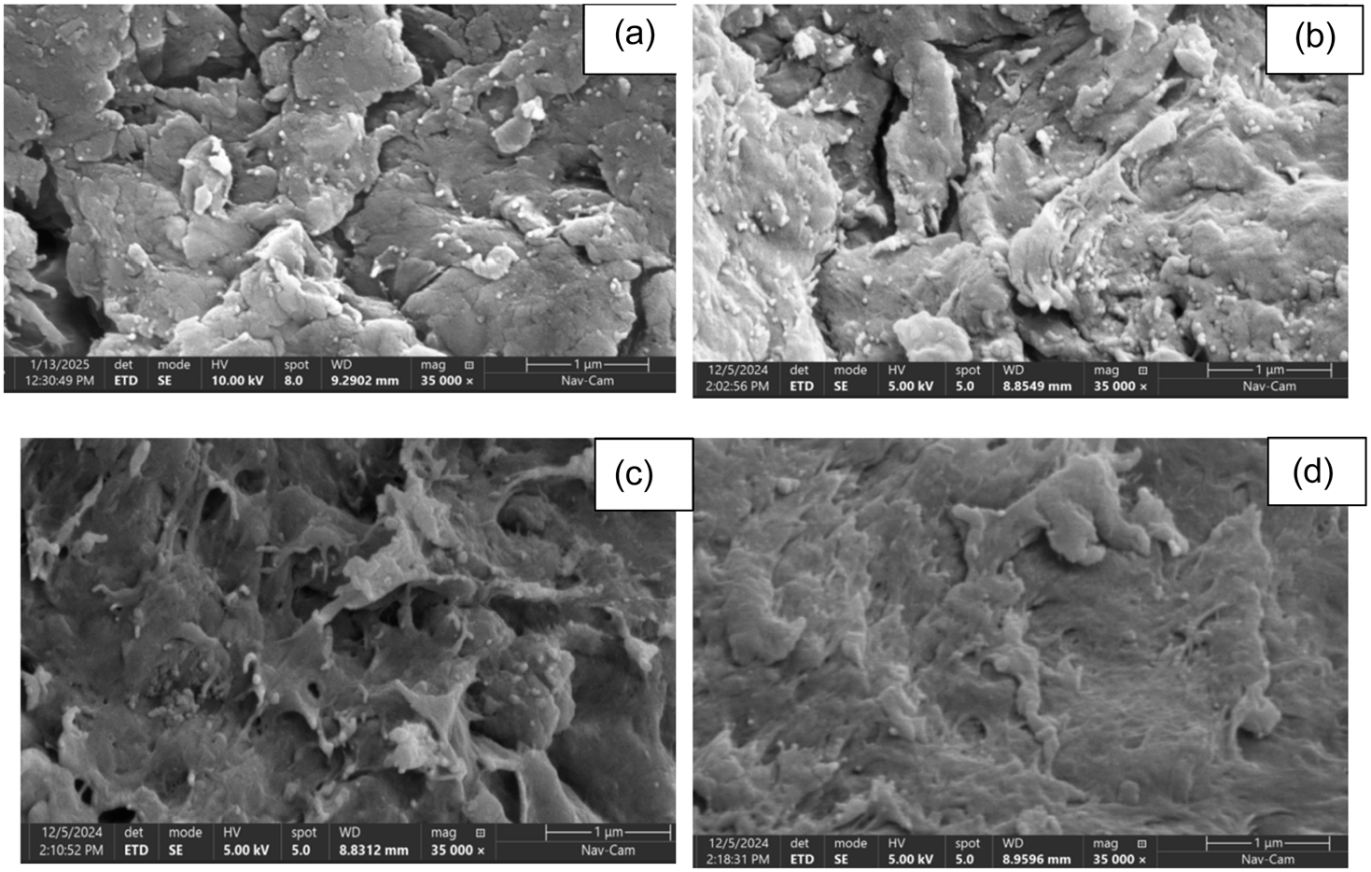
SEM images of (a) LC/PAN‐doped HCl, (b) LC/PAN‐doped H_2_SO_4_, (c) LC/PAN‐doped HCl/MB, and (d) LC/PAN‐doped H_2_SO_4_/MB.

The surface morphology and structural characteristics of the composites play a crucial role in their adsorption performance. To elucidate these aspects and their implications for dye uptake, the surface of the raw material Luffa cylindrica, LC/PAN‐doped HCl, LC/PAN‐doped H_2_SO_4_, and after adsorption of MB on LC/PAN‐doped HCl and LC/PAN‐doped H_2_SO_4_ was observed through SEM image as shown in Figure 4.

Figure S1 shows the surface of the raw Luffa. It looks homogeneous, uniform, and smooth and can clearly see the cellulose [37]. Following in situ polymerization and doping with either two different acids, HCl and H_2_SO_4_, Figure 4a,b show that the polymer successfully coated the Luffa fibers [38, 39]. The new surface looks porous and grainy, with different sized bumps and a rough, uneven texture [39]. This rougher, more complex surface suggests it has a higher surface area than the raw material Luffa cylindrica [38]. This structural alteration, particularly the increased porosity and rough texture observed in Figure 4a,b, is critical for enhancing pollutant accessibility to active sites [38].

The morphological differences observed between the two acid‐treated composites are critical and directly linked to the choice of dopant. LC/PAN‐doped HCl (Figure 4a) displays a relatively less dense and more porous coating. This morphology is consistent with the smaller monovalent chloride ions facilitating a more open polymer network formation. In contrast, the LC/PAN‐doped H_2_SO_4_ composite (Figure 4b) shows larger, more compact PAN agglomerates. This could be attributed to the larger and polyvalent nature of sulfate ions (SO_4_^2−^), which might induce different cross‐linking or aggregation patterns during polymerization, leading to a denser yet potentially more chemically active surface structure. This structural variation, particularly the differing porosity and surface roughness induced by the dopants, directly influences the accessibility of adsorption sites and the overall surface area (as reflected in BET measurements, Table 1). Furthermore, sulfate ions (SO_4_^2−^) may impart distinct charge characteristics to the surface, influencing its affinity for adsorbates and potentially leading to a more hydrophilic surface, which is crucial for adsorption in aqueous environments.

**TABLE 1:** Properties of (LC/PAN‐doped HCl) and (LC/PAN‐doped H_2_SO_4_) from BET data.

	LC/PAN‐doped HCl	LC/PAN‐doped H_2_SO_4_
BET surface area (m^2^/g)	20.7990	5.2759
Pore volume (cm^3^/g)	0.013291	0.003570
Pore size (nm)	6.0250	7.1192
Nanoparticle size (nm)	288.4748	1137.2480

After the MB dye was adsorbed, the SEM pictures (Figure 4c,d) reveal noticeable changes in the surface morphology of both materials. These changes include the appearance of new surface features or altered textures, indicating the presence of MB molecules, possibly aggregated in certain areas [35]. In particular, Figure 4d, which corresponds to the LC/PAN composite doped with H_2_SO_4_ after MB adsorption, shows a surface that appears densely coated with MB. Although this material exhibits a lower specific surface area (as shown in Table 1), the extensive surface coverage suggests a strong interaction or high affinity between the MB molecules and specific active sites on the LC/PAN‐doped H_2_SO_4_ surface. This could contribute to its adsorption performance, potentially due to favorable surface chemistry, such as enhanced hydrophilicity or the presence of specific binding sites, which may compensate for the lower overall accessible surface area compared with the LC/PAN‐doped HCl composite. These morphological observations from SEM provide direct visual evidence of the successful coating of Luffa by PAN and, critically, the subsequent uptake of MB dye onto the composite surfaces. The distinct post‐adsorption morphologies for each composite underscore the influence of the dopant acid on the composite's surface characteristics, directly impacting its interaction with the dye molecules.

As shown in Table 1, LC/PAN‐doped HCl exhibits a significantly higher BET surface area (20.7990 m^2^/g) and smaller average nanoparticle size compared to LC/PAN‐doped H_2_SO_4_ (5.2759 m^2^/g and larger nanoparticles). This seemingly counterintuitive observation, given the superior adsorption performance of the H_2_SO_4_‐doped variant, suggests that total surface area is not the sole or predominant factor governing MB adsorption in these composites. Instead, the nature and accessibility of specific active sites, which are profoundly influenced by the type of acid dopant, play a more crucial role. The denser packing and larger agglomerates observed in the SEM for the H_2_SO_4_‐doped composite (Figure 4c) might indeed lead to a lower measured BET area. However, its surface chemistry, modified by the sulfate ions, could offer a higher affinity or more effective binding configurations for MB, potentially through enhanced electrostatic interactions or specific complexation, which compensates for the reduced accessible surface area. This result highlights the complex interplay between morphology, surface chemistry, and adsorption performance driven by the choice of dopant.

**FIGURE 5:**
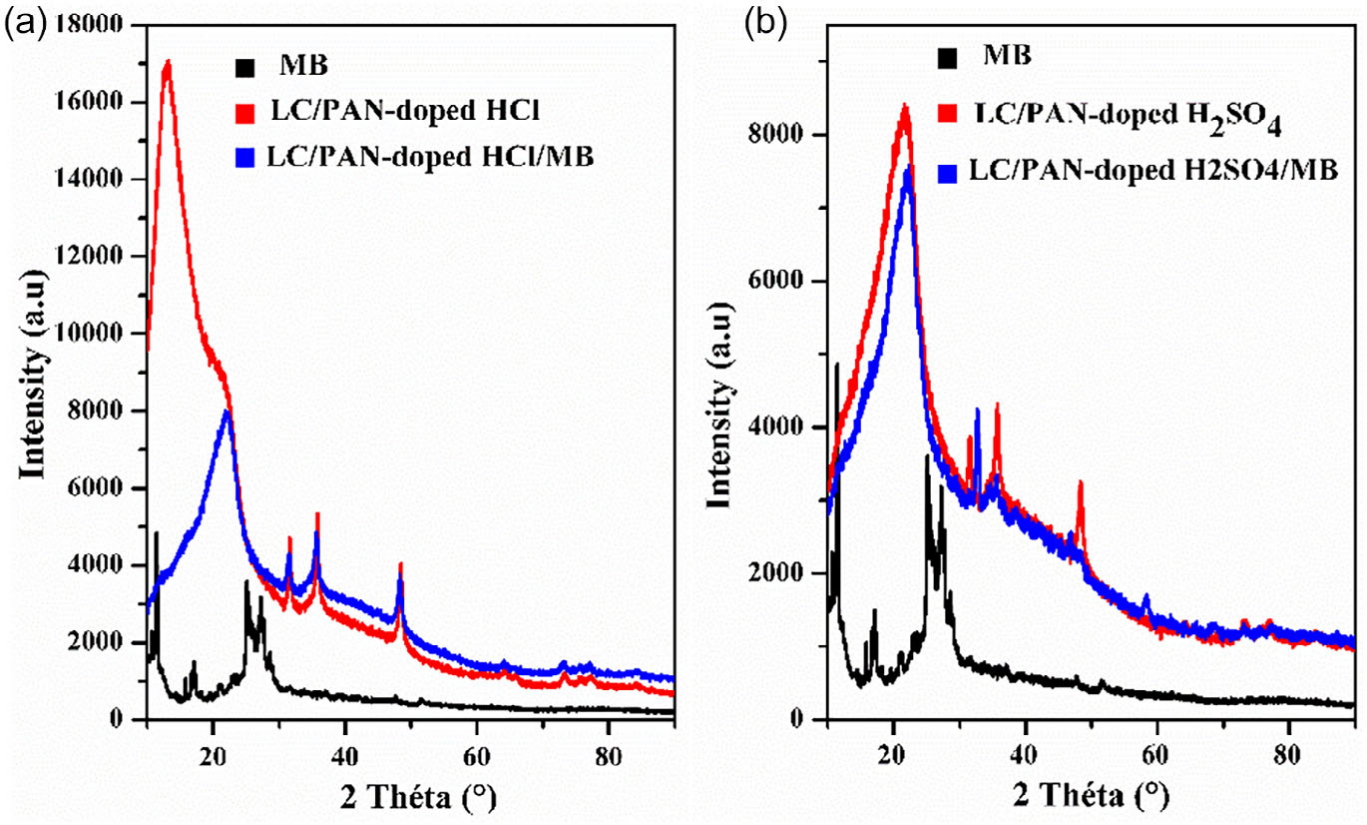
XRD spectrum of (a) LC/PAN‐doped HCl and LC/PAN‐doped HCl/MB and (b) LC/PAN‐doped H_2_SO_4_ and LC/PAN‐doped H_2_SO_4_/MB.

The X‐ray diffraction (XRD) patterns of LC/PAN composites, before and after MB adsorption, are displayed in Figure 5, which provide important insights on the structural influence of doping acids on both the polyaniline network and its interaction with the Luffa cellulosic backbone. Before adsorption, the XRD spectra of LC/PAN‐doped HCl shows broad peaks at 2*θ * ≈  20.69°, and 31.4°, while LC/PAN‐doped H_2_SO_4_ shows broad peaks at 2*θ * ≈  21.71° and 31.53°. These broad reflections are characteristic of the semicrystalline nature of emeraldine salt polyaniline, reflecting its relatively ordered chain packing [29]. The subtle shifts in these peak positions between LC/PAN‐doped HCl and LC/PAN‐doped H_2_SO_4_ are highly significant, suggesting variations in the interchain spacing and packing arrangement of the PAN polymer due to the different sizes and interactions of the counter‐ions. This indicates that the dopant acid directly influences the PAN's crystalline order and lattice parameters.

Superimposed on these PAN signals are reflections from the native cellulose Iβ structure of Luffa cylindrica fibers. A clear diffraction peak was observed at 2*θ * ≈  13.04° in the LC/PAN‐doped HCl sample, assigned to the merged 1–10 and 110 reflections of cellulose Iβ, which is a common feature for celluloses with small crystallite sizes [40, 41]. This peak's more prominent presence in the HCl‐doped sample compared to the H_2_SO_4_‐doped material could indicate differences in the degree of PAN coating uniformity or penetration into the Luffa fiber's cellulosic structure, influenced by the acid‐mediated polymerization environment. While mechanical grinding during sample preparation might expose underlying cellulose, the differential exposure of cellulosic signals suggests a distinct interaction and coverage mechanism dependent on the dopant type. The main cellulose reflection, the 200 peak, which is typically the most intense and located around 22.5° for cellulose Iβ [42], is likely overlapped and masked by the dominant broad peak of PAN in both composites. A weak peak around 2*θ * ≈  35°, assignable to the 004 reflection of cellulose [40, 41, 42, 43], further supports the presence of the cellulosic backbone. Additionally, a broad, low‐intensity hump observed at 2*θ * ≈  48° for both pristine LC/PAN composites likely represents a semi‐crystalline or weakly crystalline form within the polyaniline matrix.

Following the adsorption of MB dye (Figure 5b), notable changes occur in both the peak positions and intensities, providing crucial evidence of the dye–adsorbent interaction and its structural consequences. Generally, the intensities of the main PAN diffraction peaks decrease, accompanied by a slight shift of these peaks to higher 2*θ* values, which indicates a small reduction in the interplanar spacings upon dye binding. This disappearance is highly significant and is attributed to a more profound and specific interaction between the adsorbed MB molecules and these particular short‐range ordered regions or local conformational arrangements within the PAN structure of the LC/PAN‐doped H_2_SO_4_ composite. It is proposed that the relatively large dye molecules penetrate, interfere with, or closely associate with these specific, less‐ordered domains, leading to a significant disruption or reorganization of the polymer structure that effectively erases this diffraction feature. This distinct response, unique to the H_2_SO_4_‐doped composite, underscores how the specific counter‐ion influences the PAN's conformational flexibility and its binding capacity for the dye molecules, leading to differential structural perturbations upon adsorption. These qualitative observations from XRD are consistent with other studies on polymer‐dye interactions, where changes in diffraction patterns indicate structural rearrangements upon dye adsorption, directly impacting the composite's ability to uptake the pollutant [39]. The differential structural response between the two dopant acids highlights the crucial role of the composite's specific structural features, profoundly influenced by the acid, in facilitating effective dye uptake and dictating the interaction mechanism.

The TGA thermogram determined the thermal stability of the LC/PAN‐doped HCl and LC/PAN‐doped H_2_SO_4_ composites compared with the unbonded LC/PAN.

**FIGURE 6:**
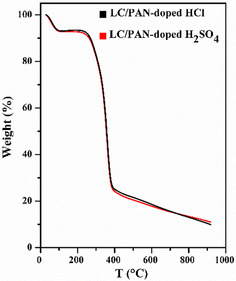
TGA curves of LC/PAN‐doped HCl and LC/PAN‐doped H_2_SO_4_.

Figure 6 shows the TGA curves of these composites. The first initial weight loss (~7%) up to 120°C is due to the evaporation of absorbed water molecules and moisture. It is evident that LC/PAN doped with the two acids exhibits higher thermal stability than unbonded LC/PAN, remaining stable up to 215°C [44]. The second major weight loss (~69%) between 200°C and 400°C can be attributed to the decomposition of the dopant bound to polyaniline, which is absent in unbonded LC/PAN [45], the degradation of polyaniline itself [39], and the decomposition of cellulose and hemicellulose from LC [46, 47]. The decomposition of lignin starts above 400°C [47, 48]. At 800°C, LC/PAN‐doped HCl and LC/PAN‐doped H_2_SO_4_ leave about 10% of the residue, higher than the residue left by unbonded LC/PAN, indicating enhanced thermal stability due to acid doping and bonding. This thermal stability, comparable to other reported polyaniline‐cellulose composites [39], is vital for the practical application of these adsorbents in diverse environmental conditions, suggesting their robustness for industrial wastewater treatment where temperature fluctuations may occur.

### 3.2 Effect of Adsorption Time

**FIGURE 7:**
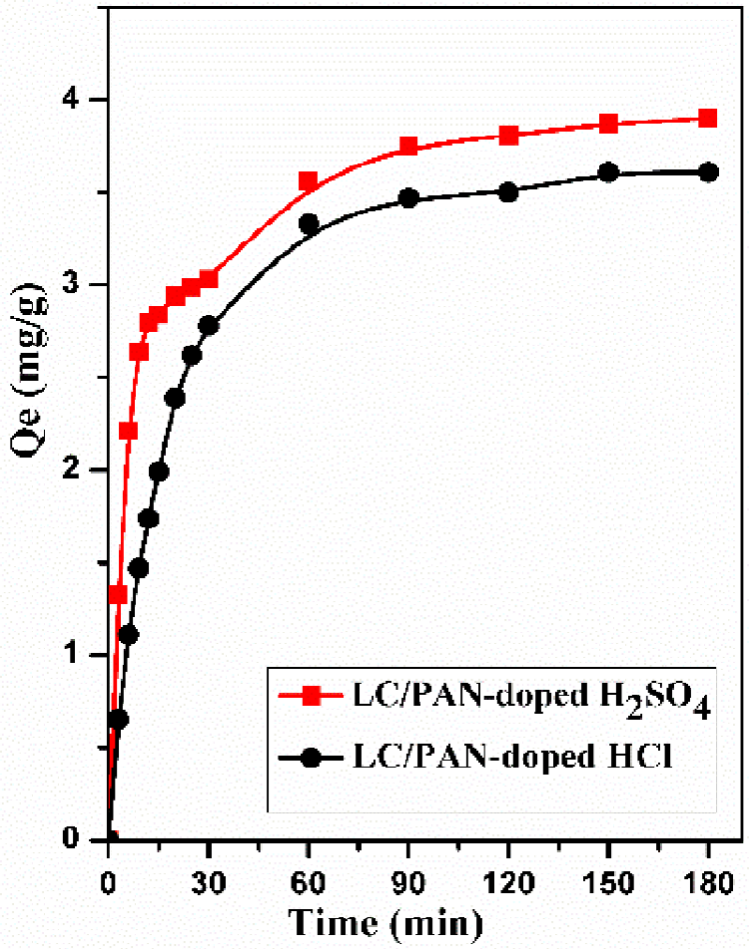
Effect of contact time on adsorption capacity Qe (mg/g) MB dye onto adsorbents. (Experimental conditions: *C*_0_ = 10 ppm, *m* = 100 mg, *v* = 500 tr/min, pH = 6, *V* = 50 mL, ambient temperature).

A crucial element in adsorption research is stirring time, and Figure 7 illustrates how contact time affects MB dye. Confirming the stirring time to achieve equilibrium in the adsorption process will be made easier by the data. The adsorption test was performed in a time range from 0 to 180 min for the two adsorbents LC/PAN‐doped HCl, and LC/PAN‐doped H_2_SO_4_.

The results showed that the increase in contact time increased the adsorption of MB on different adsorbents. The rate of adsorption was fast at the beginning of the process from 3 to 60 min, which can be explained by the number of active sites available on the surface, it was also brought on by the high forces of attraction between the dye molecules and the adsorbents and then remained slow and constant for both materials after 60 min, which can be explained by reaching saturation (almost complete occupancy of active sites), the adsorption is maximal. This rapid initial adsorption phase, followed by a slower approach to equilibrium, is a common characteristic of many adsorption processes, where initial uptake is dominated by surface binding to readily available sites, and later by slower diffusion into internal pores. The achievement of equilibrium within 60 min is highly advantageous for practical wastewater treatment, indicating efficient pollutant removal within a reasonable timeframe, which is often a critical factor for industrial applications.

### 3.3 Effect of Adsorbent Mass

**FIGURE 8:**
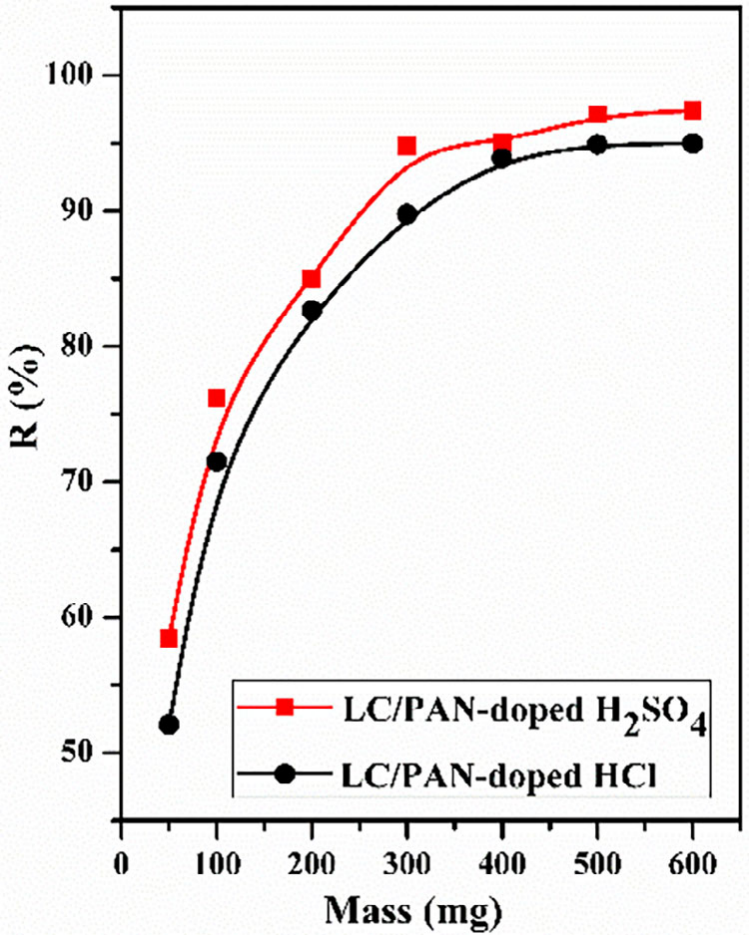
Effects of adsorbent amounts on the % removal efficiency of MB dye. (Experimental conditions: *C*_0_ = 10 ppm, *v* = 500 tr/min, pH = 6, *V* = 50 mL, ambient temperature, *t* = 120 min).

Figure 8 illustrates the impact of the adsorbent mass of LC/PAN‐doped HCl and LC/PAN‐doped H_2_SO_4_ on the % of MB dye removal rate at varying adsorbent amounts (5, 100, 200, 300, 400, 500, and 600 mg). Specifically, during adsorption, the adsorbed mass is a crucial element.

Given these findings, as the adsorbent masse increases, the % of MB dye removal rate also increases [49] (50–500 mg); this may be due to an increase in the surface area which in turn to the increase in the number of active sites, and then the % of removal remains almost constant. Also, % removal efficiency of MB was better onto LC/PAN‐doped H_2_SO_4_ than onto LC/PAN‐doped HCl. These results can be explained by the higher affinity for LC/PAN‐doped H_2_SO_4_ making many pores on the adsorbent area [50] and functional groups on the surface of the adsorbent. The observed increase in removal efficiency with adsorbent dosage is consistent with numerous studies on adsorption, as a higher adsorbent mass provides a larger surface area and more available active sites for dye binding [49, 51]. The superior removal efficiency of LC/PAN‐doped H_2_SO_4_ compared with LC/PAN‐doped HCl, despite its lower BET surface area, highlights the crucial role of surface chemistry and functional groups, which may offer a higher affinity for MB dye due to unique charge characteristics or binding interactions facilitated by the sulfate dopant [50].

### 3.4 Effect of Initial Concentration

**FIGURE 9:**
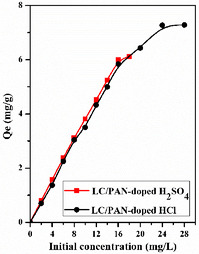
Effects of initial concentration on the equilibrium adsorption capacity. (Experimental conditions: *m* = 100 mg, *v* = 500 tr/min, pH = 6, *V* = 50 mL, ambient temperature, *t* = 120 min).

The solution's concentration has a significant impact on adsorption. Figure 9 illustrates how the initial concentration affects the percentage and adsorption capacity (mg/g) at pH‐free and room temperature.

The adsorption capacity Qe (mg/g) increased as the initial methylene blue concentration increased. As the concentration of MB increases, the number of ions in solution increases, implying a higher adsorption capacity, possibly because of a strong mass transfer driving force on both LC/PAN‐doped HCl and LC/PAN‐doped H_2_SO_4_, which had significantly more MB ions surrounding the adsorbents’ active regions. Where the quantities of MB dye adsorbed (Qe (mg/g)) increased from 0.7 mg/g to 7.28 mg/g (LC/PAN‐doped HCl), 0.85 mg/g to 6.12 mg/g (LC/PAN‐doped H_2_SO_4_). This behavior has been ascribed by earlier research to the larger concentration gradient between MB molecules and adsorbents, which speeds up dye migration onto the adsorbent surface and boosts adsorption capacity [52]. This direct relationship between initial dye concentration and adsorption capacity is a well‐established phenomenon in adsorption, driven by an increased driving force that overcomes mass transfer resistances [52, 53]. Our composites demonstrate a robust capacity to adsorb higher quantities of dye as its concentration increases, highlighting their potential for treating highly concentrated dye effluents.

### 3.5 Effect of pH

**FIGURE 10:**
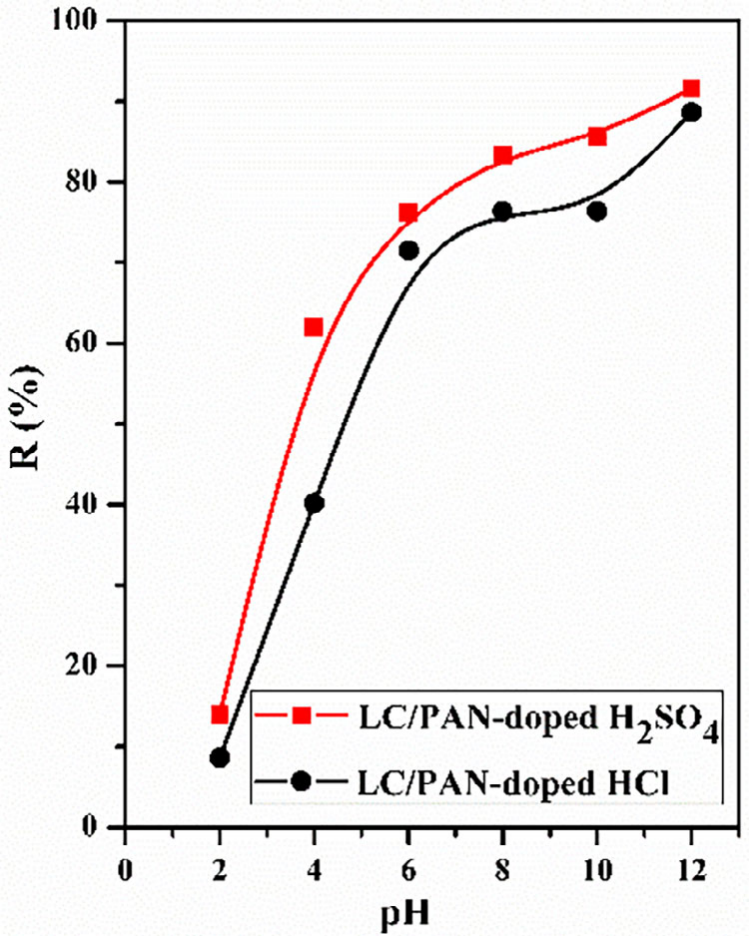
Effects of pH on the % removal efficiency of MB dye. (Experimental conditions: *m* = 100 mg, *v* = 500 tr/min, *C*_0_ = 10 ppm, *V* = 50 mL, ambient temperature, *t* = 120 min).

The pH of the solution is a critical factor influencing the adsorption process, as it dictates the surface charge of the adsorbent and the ionization state of the adsorbate. The effect of solution pH on the removal of MB by LC/PAN‐doped HCl and LC/PAN‐doped H_2_SO_4_ was investigated by varying the solution pH from 2 to 12, with an initial MB concentration of 10 ppm, using an adsorbent dose of 100 mg, and modifying the starting pH using HCl (0.1 M) and NaOH (0.1 M) solutions, as depicted in Figure 10.

**FIGURE 11:**
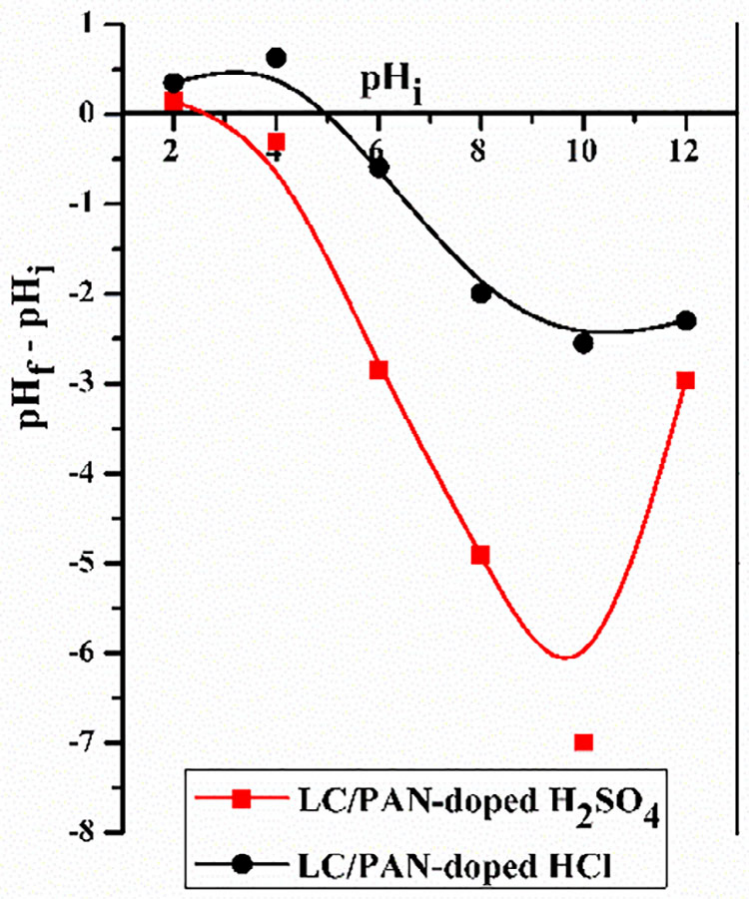
Point zero charge (pHzpc) of adsorbents.

Our results show that MB adsorption is highly dependent on pH, which is in line with several studies highlighting its role in controlling electrostatic interactions. To illustrate this, the zero point charge (ZPC), which corresponds to the pH at which the net surface charge of the adsorbent is zero, was determined (Figure 11).

A crucial finding from our study is the significant difference in pHzpc between the two composites: LC/PAN‐doped HCl exhibits a pHzpc of 5.04, while LC/PAN‐doped H_2_SO_4_ shows a notably lower pHzpc of 2.5. This disparity directly reflects the distinct protonation capabilities and the nature charge of the counter‐ion incorporated during the doping process, profoundly impacting the composite's surface chemistry.

Consequently, at pH  <  pHzpc, the surface of the adsorbents is positively charged due to the protonated amino groups of PAN [39]. In this acidic range, the adsorption of the MB cationic dye is hindered by strong electrostatic repulsion and competition from H^+^ ions [51, 52, 53, 54]. Conversely, as the pH value increases (pH  >  pHzpc), the adsorbent surfaces become increasingly negatively charged due to the deprotonation of functional groups like amines and imines, as well as the inherent hydroxy groups from Luffa [55]. This enhanced negative charge promotes a strong electrostatic attraction with the positively charged MB dye, increasing the adsorption capacity [36]. The lower pHzpc of LC/PAN‐doped H_2_SO_4_ implies that its surface achieves a negative charge at a much lower pH, offering a broader and more favorable pH range for effective electrostatic interaction with MB compared to LC/PAN‐doped HCl. This explains, in part, the superior performance observed for the H_2_SO_4_‐doped composite across various pH conditions.

### 3.6 Effect of Temperature

**FIGURE 12:**
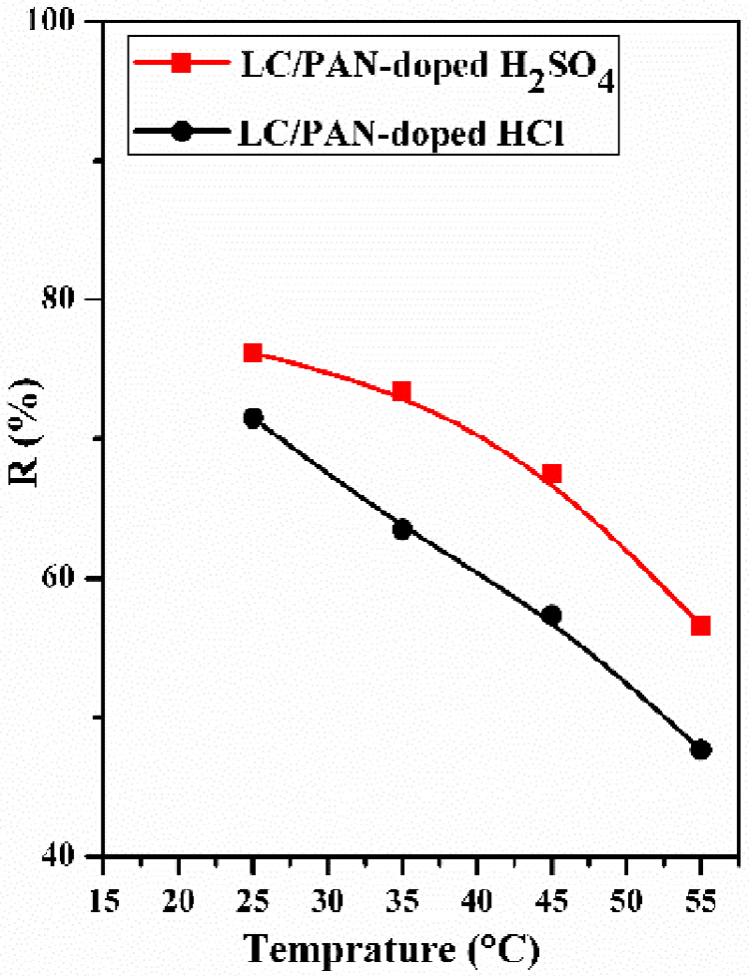
Effects of temprature on the % removal efficiency of MB dye. (Experimental conditions: *m* = 100 mg, *v* = 500 tr/min, *C*_0_ = 10 ppm, *V* = 50 mL, pH = 6, *t* = 120 min).

Since most textile dye effluents are produced at relatively high temperatures [56], the effect of temperature is another important element in the adsorption process and is also an indicator of the type of adsorption [57]. The adsorption of MB was conducted at different temperatures (25°C, 35°C, 45°C, and 55°C) with 100 mg of LC/PAN‐doped HCl and LC/PAN‐doped H_2_SO_4_, as shown in Figure 12.

It can be seen that the MB adsorption significantly decreased from 71.5% to 47.66% for LC/PAN‐doped HCl and from 76.2% to 56.6% for LC/PAN‐doped H_2_SO_4_ with the rising of temperature from 25°C to 55°C, which may be ascribed to the surface deformation, decreasing the sorptive forces between MB and the active sites. Referring to the weakening of the sorptive forces on the adsorbent surfaces [57, 58]. This inverse relationship between temperature and adsorption capacity confirms the exothermic nature of the MB adsorption process on LC/PAN composites, aligning with similar observations for physical adsorption on various materials where increased kinetic energy of adsorbate molecules at higher temperatures reduces their affinity for the adsorbent surface.

### 3.7 Thermodynamic Parameters

Thermodynamic parameters are evaluated by temperature effect results on LC/PAN‐doped HCl and LC/PAN‐doped H_2_SO_4_ for adsorption MB in aqueous solution at different temperatures. Standard enthalpy (Δ*H*°, kJ/mol), standard free energy (Δ*G*°, kJ/mol), and standard entropy (Δ*S*°, kJ/mol.K) have been calculated by the following equations:

(3) $$\Delta G^{\circ }=\Delta H^{\circ }-T\Delta S^{\circ }$$

(4) $$\Delta G^{\circ }=-RT\ln\left(K_{c}\right)$$

(5) $$\ln\left(K_{c}\right)=\frac{\Delta S^{\circ }}{R}-\frac{\Delta H^{\circ }}{TR}$$

where *T* is the temperature (K), *R* is the gas constant (8.314 J/mol.K), and *K*_c_ is the distribution coefficient

The parameter *K*_c_ calculated by the following equation:

(6) $$K_{c}=\frac{Q_{e}}{C_{e}}$$

where *Q*_e_ is the solid phase concentration (mg/g) and *C*_e_ is the equilibrium concentration of dye on the adsorbent (mg/L).

**FIGURE 13:**
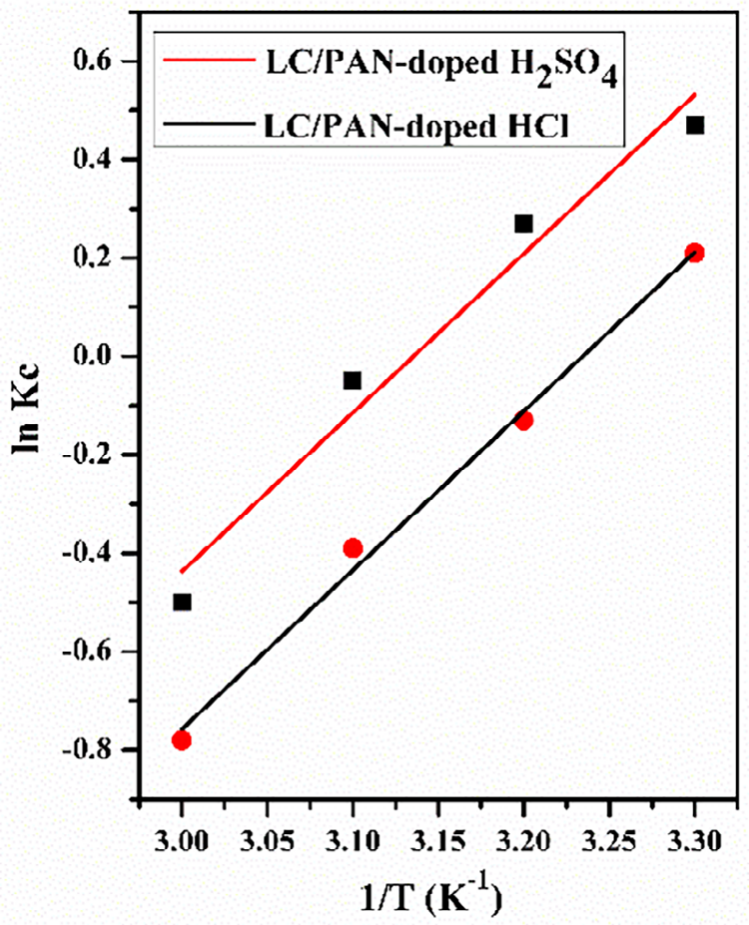
Thermodynamic plot of ln *K*_c_ vs. 1/*T*.

The values of Δ*S*° and Δ*H*° were obtained from the slope and intercept of the plot ln *K*_c_ vs (1/*T*) as seen in Figure 13. The results are in Table 2.

**TABLE 2:** Thermodynamic adsorption parameters.

Adsorbents	Temperature, K	Δ*S*, kJ/mol.K	Δ*H*, kJ/mol	Δ*G*, kJ/mol	*R* ^2^
LC/PAN‐doped HCl	298	−0.08	−26.76	−2.92	0.98432
	308			−2.12	
	318			−1.32	
	328			−0.52	
LC/PAN‐doped H_2_SO_4_	298	−0.08	−26.85	−3.01	0.97091
	308			−2.61	
	318			−1.41	
	328			−0.61	

According to the results, the negative values of the free enthalpy (Δ*G* < 0) allow us to say that the MB adsorption reaction on both adsorbents is feasible and spontaneous. Besides, negative enthalpy values (Δ*H*) confirm that adsorption is exothermic; these values are less than 40 kJ/mol, suggesting that the system is physisorption [59, 60, 61], and this result suggests weak forces of attraction at the interface between the adsorbent and solution, where the primary forces are hydrogen bonds, electrostatic interactions, van der Waals forces, and π–π interactions [62, 63]. The probability of thermodynamically advantageous adsorption is shown by a negative value of Δ*S* [64, 65], which also indicates a decrease in randomness at the adsorbent/solution interface during adsorption [63, 66]. These thermodynamic parameters are consistent with MB adsorption onto various polymer and cellulose‐based composites, generally indicating spontaneous, exothermic, and physisorption‐dominated processes [62]. The decrease in randomness (negative Δ*S*) at the adsorbent‐solution interface is typical for adsorption mechanisms involving the organization of adsorbate molecules onto the adsorbent surface.

### 3.8 Adsorption Isotherms

**FIGURE 14:**
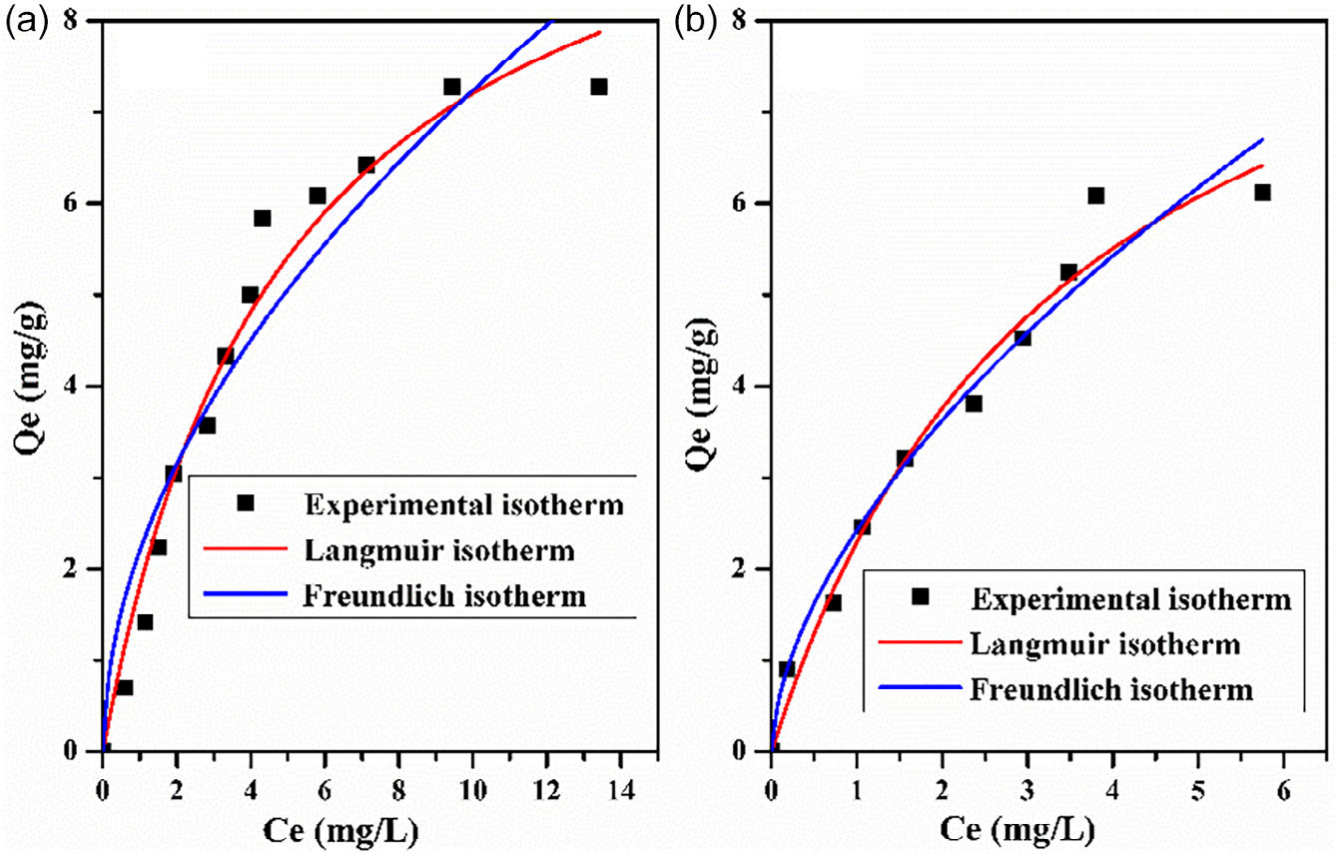
Adsorption isotherm for MB onto: (a) LC/PAN‐doped HCL and (b) LC/PAN‐doped H_2_SO_4_.

Adsorption isotherms are essential for comprehending the adsorption mechanism and how MB molecules interact with adsorbents [50], which are also utilized for system description [61] and determining the type of mechanism. Besides, it is used to determine the adsorption capacity of the adsorbates (MB) on the adsorbents. Figure 14 displays the equilibrium adsorbed quantity (*Q*_e_: mg/g) variation as a function of equilibrium concentration (*C*_e_: mg/L).

In this study, adsorption equilibrium is analyzed by applying the Langmuir and Freundlich models, which are commonly used by researchers due to their simplicity for studying adsorption isotherms systems.

**FIGURE 15:**
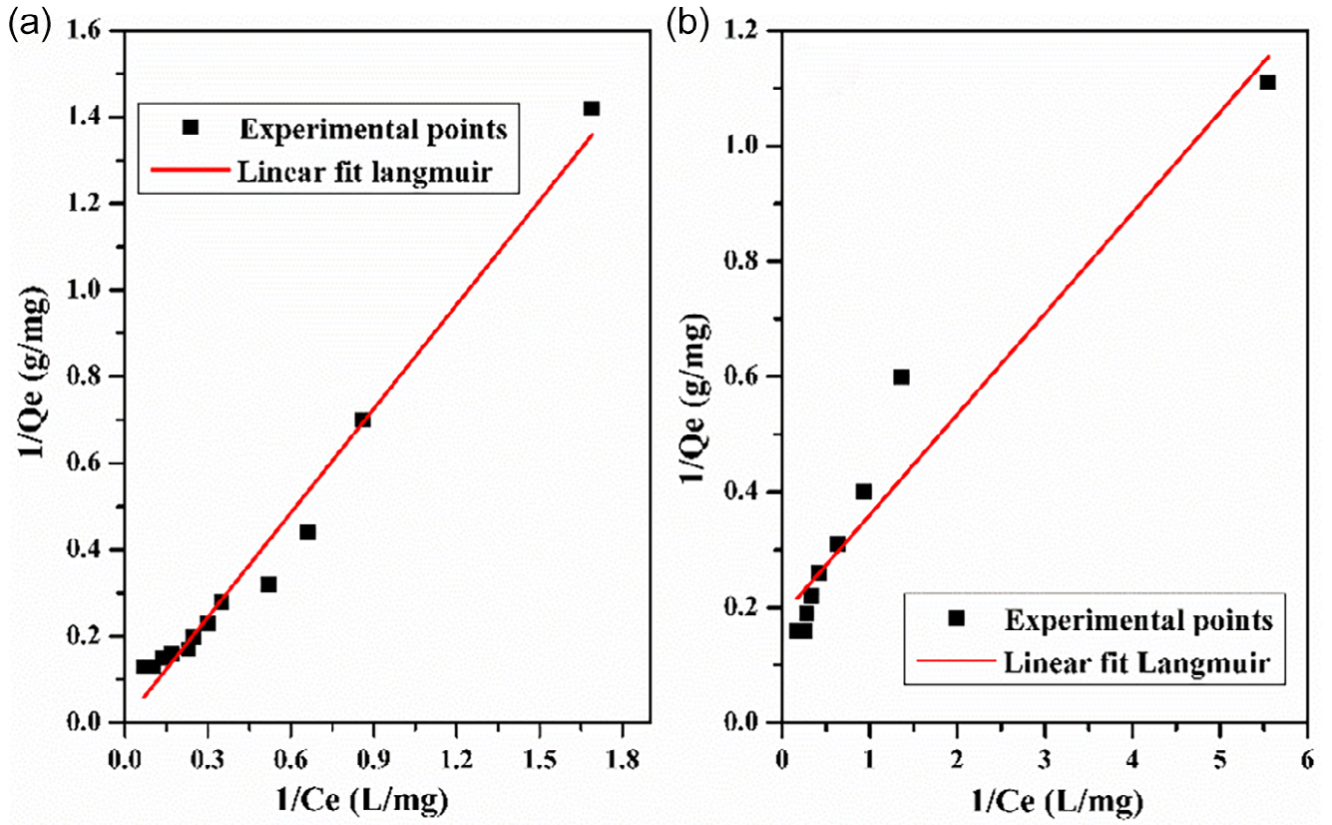
Langmuir isotherms for the adsorption of MB on: (a) LC/PAN‐doped HCL and (b) LC/PAN‐doped H_2_SO_4_.

The Langmuir isotherm assumes that adsorption is monolayer and homogeneous [39, 67, 68], that adsorbate adsorption takes place on a surface with specific sites [69], and that adsorbed molecules do not engage in lateral interactions [50]. The Langmuir model is defined by the following equation (Langmuir 1916):

(7) $$\frac{1}{q_{e}}=\frac{1}{Q_{\max}}+\frac{1}{K_{L}Q_{\max}C_{e}}$$

where *Q*_e_ (mg/g) represents the amount of dye adsorbed on the adsorbents at equilibrium, *C*_e_ (mg/L) stands for the equilibrium of adsorbate concentration, *Q*_max_ (mg/g) is the maximum adsorption capacity values, and *K*_L_ (L/mg) is the adsorption equilibrium constant, which depends on temperature. The values of *Q*_max_ and *K*_L_ are determined from the intersection and the slope of the straight line. Figure 15 displays the linear plot of the Langmuir isotherm of adsorbents; the parameter's value and the correlation coefficient are represented in Table 3.

**TABLE 3:** isotherm constants for adsorption of methylene blue onto LC/PAN‐doped HCL and LC/PAN‐doped H_2_SO_4_.

Adsorbents	Langmuir				Freundlich		
	*K*_L_, L/mg	*Q*_max_, mg/g	*R*_L_	*R* ^2^	$\frac{1}{n}$	*K*_F_, L/g	*R* ^2^
LC/PAN‐doped HCl	0.20	10.76	0.33	0.97	0.52	2.20	0.92
LC/PAN‐doped H_2_SO_4_	0.29	10.30	0.26	0.97	0.58	2.43	0.96

The essential feature of the Langmuir isotherm is *R*_L_, called the equilibrium parameter, which is calculated by the following equation:

(8) $$R_{L}=\frac{1}{1+K_{L}C_{i}}$$

where *C*_i_ represents the initial concentration of MB (mg/L) in the solution. The value of *R*_L_ indicates the type of isotherm, where *R*_L _= 1 is linear and 0 < *R*_L _< 1 is favorable.

The Freundlich isotherm assumes that adsorption occurs on a heterogeneous surface and is not restricted to the formation of a monolayer [70, 71, 72]. Instead, it suggests that adsorption sites possess different affinities and energies of interaction with the adsorbate molecules [50]. This empirical model is expressed by the following equation (Freundlich, 1906):

(9) $$\ln Q_{e}=\ln K_{f}+\frac{1}{n}\ln C_{e}$$

where *K*_f_ (L/g) represents the Freundlich constant related to binding energy and n is the heterogeneity factor, which $\frac{1}{n}$ value indicates the type of isotherm, where $\frac{1}{n}=0$ is Irreversible, $0<\frac{1}{n}<1$ is Favorable and $\frac{1}{n}>1$ is Unfavorable.

**FIGURE 16:**
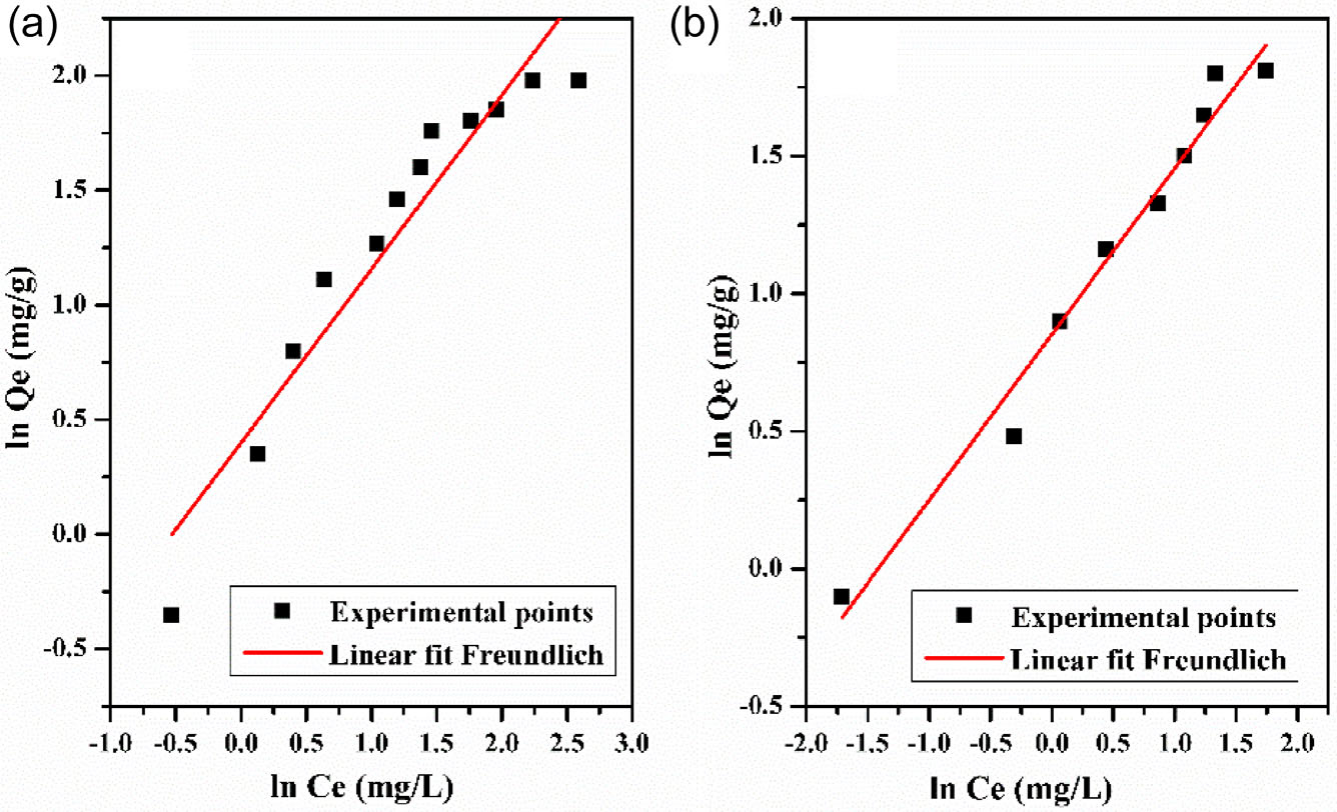
Freundlich isotherms for the adsorption of MB on: (a) LC/PAN‐doped HCL and (b) LC/PAN‐doped H_2_SO_4_.

The parameters of Freundlich *K*_f_ and n are determined from the plot of ln *Q*_e_ (mg/g) versus ln *C*_e_ (mg/L). Figure 16 represents the plot of the Freundlich isotherm of adsorbents. The value of these parameters and the correlation coefficient are displayed in Table 3.

From Figure 14, it was observed that *Q*_e_ increases with increasing *C*_e_ until equilibrium is reached, corresponding to a saturation of the adsorption sites. The interaction between MB and adsorbents was enhanced by the rise in *C*_e_, and it provided the required push to get beyond the obstacles to MB mass transfer between the aqueous and solid phases [53]. The isotherm obtained is of type L according to Giles’ classification [65, 73].

From Table 3, it was seen that the Langmuir isotherm fits the experimental data perfectly (*R* ^2^ = 0.97) compared with the Freundlich in both adsorbents, which confirms the monolayer coverage and the homogeneous distribution of active sites onto adsorbents’ surfaces. Additionally, the favorable uptake of the methylene blue process is confirmed by the value in the range of 0–1 at all initial dye concentrations. While the determination coefficients for Freundlich and Langmuir models were quasi‐identical for LC/PAN‐doped H_2_SO_4_, the slightly higher Langmuir constant for LC/PAN‐doped H_2_SO_4_ (0.29 L/mg) compared with LC/PAN‐doped HCl (0.20 L/mg) indicates a better affinity for MB molecules [67]. The excellent fit to the Langmuir model for both composites confirms monolayer adsorption. However, the slightly higher affinity for LC/PAN‐doped H_2_SO_4_ (0.29 L/mg) compared with LC/PAN‐doped HCl (0.20 L/mg), despite its lower BET surface area, further reinforces the notion that the specific surface chemistry and binding sites, engineered by the sulfate dopant, are more influential than sheer surface availability in determining the adsorption strength. This suggests that the sulfate dopant may facilitate a more favorable orientation or stronger electrostatic interaction between the MB molecules and the active sites on the adsorbent surface, potentially due to altered charge distribution or exposed functional groups, thereby compensating for the lower overall surface area.

Table 4 compares the findings of this investigation with earlier research on the maximum adsorption capacity of different low‐cost adsorbents for MB dye in aqueous solution. This comparative analysis demonstrates that our LC/PAN composites exhibit a competitive adsorption performance for MB dye, often surpassing or being comparable to other low‐cost adsorbents reported in the literature. This highlights the significant potential of our bio‐based and cost‐effective materials as viable and environmentally sound alternatives for wastewater treatment. Furthermore, it explicitly suggests that the composite structure and surface chemistry, profoundly influenced by the doping acid, play a key role in achieving these effective adsorption capacities and should be considered paramount in designing optimized adsorbents. Moreover, their relevance for sustainable development and cost‐effective solutions in regions with limited resources is particularly noteworthy.

**TABLE 4:** Previously reported maximum adsorption capacity of various low‐cost adsorbents for methylene blue.

Material	Adsorbate	*Q*_max_, mg/g	pH	T, °C	Source
Sawdust cherry	MB	39.84	—	20°C	[74]
sawdust pitch‐pine	MB	27.78	—	20°C	[74]
sawdust oak	MB	29.50	—	20°C	[74]
Beech sawdust untreated	MB	9.78	—	—	[75]
Cel‐g‐PAni	MB	1.58	8	—	[37]
Beech sawdust pretreated	MB	13.02	—	23°C	[75]
PAN‐EB powder	MB	44.41	8	30°C	[35]
PAN powder	MB	11.80	6	30°C	[76]
PAN nanotubes	MB	9.20	6	25°C	[77]
Tomato stemc AC	MB	4.15	—	—	[78]
Eggshell	MB	0.80	7	25°C	[79]
LC/PAN‐doped HCl	MB	10.76	6	25°C	This study
LC/PAN‐doped H_2_SO_4_	MB	10.30	6	25°C	This study

### 3.9 Kinetics Studies

The mechanics and characteristics of adsorption are explained by adsorption kinetic models, describing the rate at which adsorbate molecules move out of the liquid phase and attach to the surface of the adsorbent. These models are crucial for understanding and optimizing adsorption processes and predicting the performance of adsorptive materials. This dynamic process is influenced by factors such as the adsorbent and adsorbate's characteristics, system temperature, and adsorbate concentration. Understanding adsorption kinetics is particularly important in environmental remediation. To investigate the kinetic mechanism of the MB adsorption process onto LC/PAN‐doped HCl and LC/PAN‐doped H_2_SO_4_ adsorbents, three different models the Pseudo‐first‐order, Pseudo‐second‐order, and intraparticle diffusion models were employed to fit the experimental adsorption data.

**FIGURE 17:**
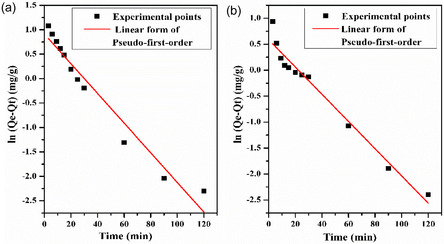
Linear plot of first‐order kinetic model for the removal of MB on: (a) LC/PAN‐doped HCl and (b) LC/PAN‐doped H_2_SO_4_.

The kinetics of dye adsorption have been extensively predicted using the pseudo‐first‐order kinetic model. The pseudo‐first‐order model was described by Lagergren in a linear form as [80]:

(10) $$\ln\left(Q_{e}-Q_{t}\right)=\ln Q_{e}-K_{1}t$$

where *K*_1_ (min^−1^) the pseudo first‐order adsorption equilibrium rate constant, *Q*_*t*_ and *Q* (mg/g) are the amount adsorbed at time *t* and at equilibrium. The slope and intercept of the plots ln (*Q*_e _− *Q*_*t*_) versus *t* (Figure 17) were used to determine *K*_1_ and *Q*_e_. Table 5 shows the results of the various first‐order‐model values.

**TABLE 5:** Kinetics parameters of pseudo‐first‐order, pseudo‐second‐order, and intra‐particle diffusion models for the adsorption of MB.

Adsorbant	Pseudo first‐order			Pseudo second‐order			Intraparticle diffusion		
	*Q*_e_, mg/g	*K*_1_, min^−1^	*R* ^2^	*Q*_e_, mg/L	K_2_, g/mol.min	*R* ^2^	*K*_id_, mg/g.min^1/2^	*C*	*R* ^2^
LC/PAN‐doped HCl	2.49	0.03	0.96	4.13	0.02	1	0.24	0.94	0.85
LC/PAN‐doped H_2_SO_4_	1.82	0.03	0.98	3.98	0.04	0.97	0.17	1.97	0.79

**FIGURE 18:**
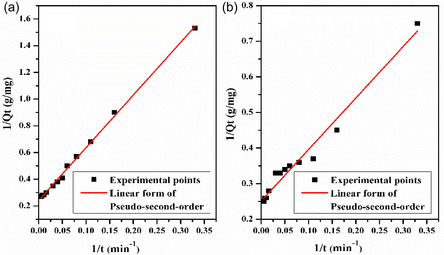
Linear plot of second‐order kinetic model for the removal of MB on: (a) LC/PAN‐doped HCl and (b) LC/PAN‐doped H_2_SO_4_.

The second‐order‐model is a valuable tool for describing adsorption kinetics, as it is based on the solid‐phase adsorption capacity, making it particularly useful for modeling chemisorption processes [81]. This kinetic model assumes that the rate of adsorption is proportional to the square of the number of unoccupied sites on the surface of the adsorbent. The equation is as follows [82]:

(11) $$\frac{1}{Q_{t}}=\frac{1}{Q_{e}}+\frac{1}{{Q_{e}}^{2}K_{2}}$$

where *K*_2_ (g/mol min) the adsorption rate constant of the pseudo‐second‐order equation; the amounts adsorbed at time *t* and at equilibrium are denoted by *Q*_*t*_ and *Q*_e_ (mg/g) respectively. The slope and intercept of the plots $\frac{1}{Q_{t}}$ versus $\frac{1}{t}$ (Figure 18) was used to determine the second‐order rate constants *K*_2_ and *Q*_e_ (Table 5).

**FIGURE 19:**
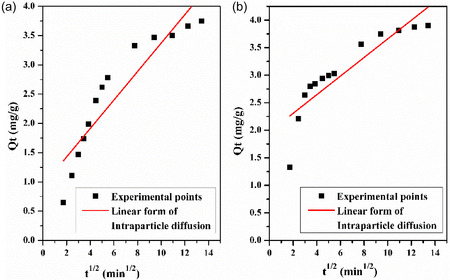
Linear plot of Intra‐particle diffusion kinetic model for the removal of MB on: (a) LC/PAN‐doped HCl and (b) LC/PAN‐doped H_2_SO_4_.

Intraparticle diffusion is a critical factor in heterogeneous adsorption processes. Refer to the transport of adsorbate molecules within the pores of adsorbents, which is often the rate‐limiting step in adsorption; the intraparticle diffusion rate constant *K*_id_ is obtained by the Mories–Weber and Morris equation [83]:

(12) $$Q_{t}=K_{\mathrm{id}}t^{1/2}+C$$

where *Q*_*t*_ (mg/g) is the amount adsorbed at time *t*, *K*_id_ (mg/g.min^1/2^) is the intraparticle diffusion rate constant, and *C* is a constant. The plots of *Q*_*t*_ versus *t* ^1/2^ for LC/PAN‐doped HCl and LC/PAN‐doped H_2_SO_4_ are shown in Figure 19. *K*_id_ and *C* are estimated from the slope and intercept, and the parametric values are given in Table 5.

To identify the most appropriate kinetic model, a comprehensive evaluation was performed based on two key criteria: the linear regression correlation coefficient (*R* ^2^) and the agreement between the experimental equilibrium adsorption capacity (*Q*_e,__exp_) and the model‐calculated capacity (*Q*_e,__cal_). The kinetic parameters for both adsorbents are summarized in Table 5.

For the LC/PAN‐doped HCl adsorbent, the pseudo‐second‐order model demonstrated a superior fit. It yielded a perfect correlation coefficient (*R* ^2^ = 1), higher than that of the pseudo‐first‐order model (*R* ^2^ = 0.96). More significantly, the *Q*_e,__cal_ value from the pseudo‐second‐order model (4.13  mg/g) was in excellent agreement with the experimental value (*Q*_e,__exp_  ≈  3.6 mg/g), whereas the pseudo‐first‐order model's calculated capacity (2.49 mg/g) showed a substantial deviation.

A similar analysis for the LC/PAN‐doped H_2_SO_4_ adsorbent revealed that despite the pseudo‐first‐order model having a higher *R* ^2^ value (0.98 vs. 0.97), it failed to accurately represent the physical process. The *Q*_e,__cal_ from the pseudo‐first‐order model (1.82  mg/g) was drastically different from the experimental capacity (*Q*_e,__exp_  ≈  3.8 mg/g). In contrast, the pseudo‐second‐order model provided a *Q*_e,__cal_ of 3.98 mg/g, which is an almost perfect match to the experimental result.

Therefore, based on the crucial agreement between calculated and experimental adsorption capacities, the pseudo‐second‐order model is the most suitable for describing the adsorption kinetics for both composites. This consistent fit to the pseudo‐second‐order model suggests that the rate‐limiting step for MB adsorption on both LC/PAN materials is likely chemisorption. This process involves the formation of chemical bonds through the sharing or exchange of electrons between the functional groups on the adsorbent surfaces and the cationic dye molecules.

From Figure 19, the curve shows the appearance of three stages in the adsorption of MB onto LC/PAN‐doped HCl and LC/PAN‐doped H_2_SO_4_: The first stage Expressing an initial linear rise portion indicates the effect of the boundary layer [71], which is the diffusion of MB from the solution to the outside surface and then the diffusion into the large pores where occupied the active sites in particular appropriate pores of both adsorbents [84].

The second stage represents a less steep portion of the adsorption process. This is due to the effect of intraparticle diffusion [71], where MB molecules must diffuse within the particles to reach the remaining adsorption sites. Over time, as more sites are occupied, intraparticle diffusion becomes the rate‐limiting step of the adsorption. Additionally, the number of available active sites decreases, and steric hindrance from already adsorbed molecules increases, further slowing down the process and resulting in this second linear portion of the adsorption curve.

The last stage is the plateau portion which corresponding to the equilibrium, due to the diffusion of internal particles was slowed down by the concentration of MB remaining in the solution. Moreover, the intersection values provide an indication of the thickness of the boundary layer; the larger the intersection values, the more effective the boundary layer is [85, 86]. The consistent fit to the pseudo‐second‐order model for both LC/PAN composites suggests that chemisorption is the rate‐limiting step. The slight differences in rate constants and calculated capacities between the two composites are likely influenced by the distinct surface energetics and accessibility of reactive sites, which are modulated by the respective acid dopants.

### 3.10 Regeneration of LC/PAN‐Doped HCl and LC/PAN‐Doped H_2_SO_4_

Regeneration of the adsorbent is essential since it lowers the costs related to the adsorption process. Therefore, it is essential for sustainability and economic viability.

**FIGURE 20:**
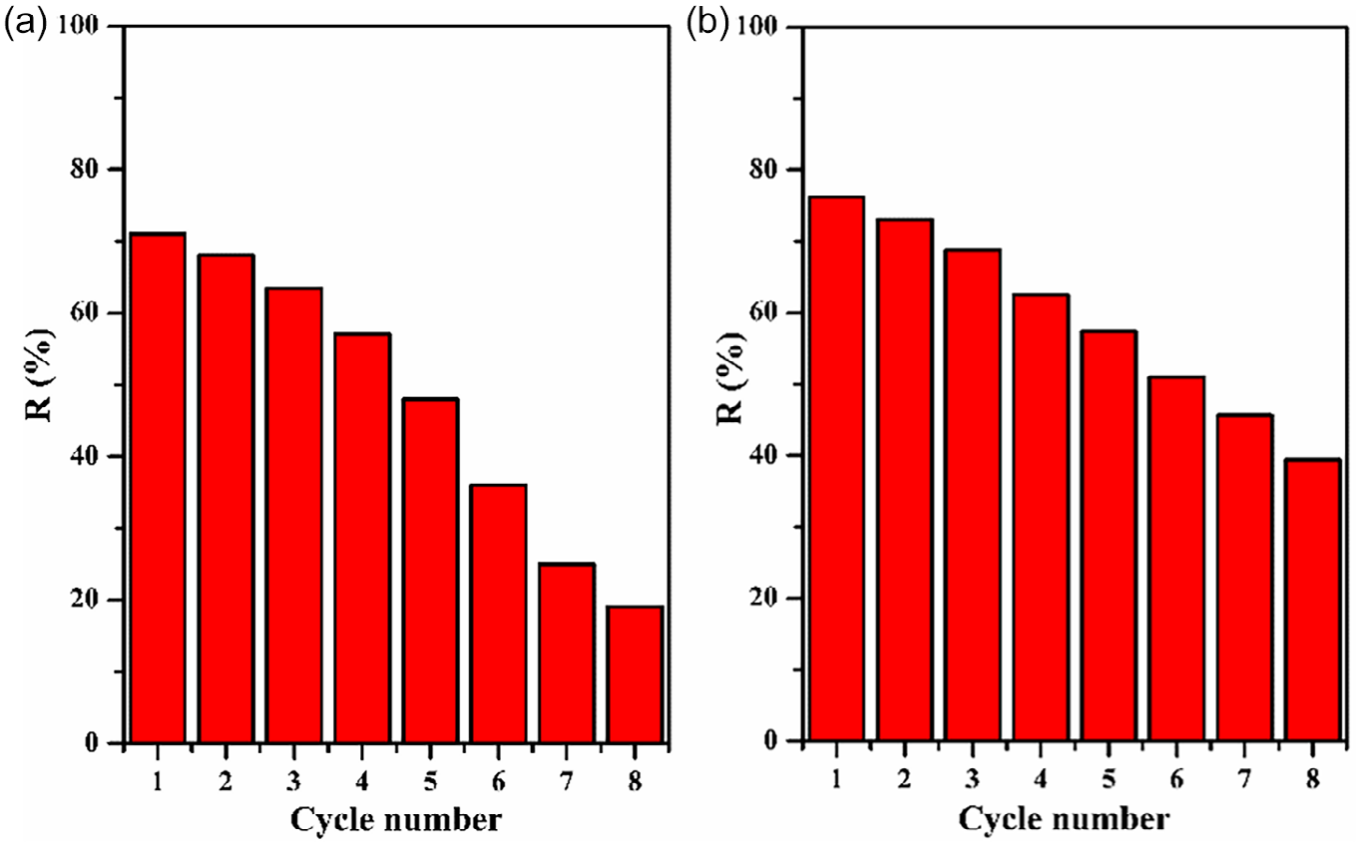
Regeneration of the (a) LC/PAN‐doped HCl, (b) LC/PAN‐doped H_2_SO_4_ (experimental conditions: *m* = 100 mg, *v* = 500 tr/min, *C*_0_ = 10 ppm, *V* = 50 mL, *t* = 180 min).

Selecting the right solvent is essential to recovering MB for further adsorptions; in this study, the ethanol solvent was chosen because the MB adsorbate is a polar dye that makes ethanol effective in removing polar adsorbates. In addition, ethanol is less toxic than other solvents and is typically easily accessible and reasonably priced. In Figure 20, the LC/PAN‐doped HCl and LC/PAN‐doped H_2_SO_4_ regeneration efficiencies following varying numbers of regeneration cycles are displayed. As can be seen, the regeneration efficiency decreased along with the regeneration cycles from 71.05% to 19.01% for LC/PAN‐doped HCl and from 76.2% to 39.38% for LC/PAN‐doped H_2_SO_4_. It was because, during each regeneration process, ethanol may not completely remove the adsorbate from the adsorbent. This results in a reduced surface area available for further adsorption due to the accumulation of adsorbates. Furthermore, repeated regeneration cycles can cause the adsorbent to lose its activity or structural integrity. The demonstrated reusability for up to eight cycles, despite a diminishing efficiency, is crucial for the economic viability and environmental sustainability of the adsorbent. While some reduction in efficiency is common for regenerated adsorbents, the ability to recover a significant portion of adsorption capacity over multiple cycles highlights the practical potential of these LC/PAN composites for repeated use in wastewater treatment applications.

### 3.11 Adsorption Mechanism

The adsorption of methylene blue onto LC/PAN composites is mainly governed by the surface chemical properties of polyaniline and the nature of the acid dopant. Although the LC/PAN–HCl composite exhibited a higher BET surface area, the LC/PAN–H_2_SO_4_ composite showed higher adsorption capacity, indicating that adsorption is controlled primarily by surface interactions rather than surface area alone.

This behavior can be attributed to the effect of sulfate ions (SO_4_^2−^), which induce stronger protonation of the polyaniline chains and modify the surface chemical environment. The presence of sulfate ions enhances the formation of adsorption‐active sites and improves the interaction between the composite surface and methylene blue molecules.

In addition, the aromatic structure of polyaniline promotes interaction with methylene blue molecules through π–π interaction. Electrostatic interaction and surface affinity also contribute to the adsorption process. The luffa fibers provide a porous structure that facilitates dye diffusion and improves accessibility to active sites.

These results indicate that the adsorption performance depends mainly on the surface chemical characteristics of the composite, and that the type of acid dopant plays an important role in improving adsorption efficiency.

## 4 Conclusion

This study successfully elucidated the efficacy of novel bio‐based and low‐cost LC/PAN composite adsorbents for methylene blue dye removal, a dangerous pollutant, with a detailed focus on the profound impact of acid dopants (HCl and H_2_SO_4_) on their structural properties and subsequent adsorption behavior. Comprehensive characterization confirmed successful composite synthesis and revealed distinct structural, morphological, and surface chemical attributes imparted by each acid. Significantly, while LC/PAN‐doped HCl exhibited a higher total surface area, the LC/PAN‐doped H_2_SO_4_ variant consistently demonstrated superior adsorption performance, underscoring that surface chemistry and the nature of binding sites, as modulated by the dopant, are more critical determinants of efficiency than overall porosity.

The adsorbents exhibited rapid kinetics, best described by the pseudo‐second‐order model, suggesting a chemisorption‐dominated process. Favorable monolayer adsorption capacities were observed, consistent with the Langmuir model. The adsorption was spontaneous, exothermic, and highly influenced by pH, with the lower pHzpc of the H_2_SO_4_‐doped composite contributing to its enhanced performance in alkaline conditions. Furthermore, both materials demonstrated reusability for up to eight regeneration cycles.

These findings provide critical insights into the structure‐property‐performance relationship in bio‐based polymer composites, emphasizing how judicious selection of the doping acid can be a powerful tool for tailoring the material's physicochemical properties for optimized and selective adsorption applications. This work highlights the great potential of LC/PAN composites as economically viable and environmentally sound options for treating dye‐contaminated wastewater, offering a sustainable approach to resource valorization and pollution control, particularly relevant for addressing water quality challenges in Africa and other developing regions. Our contribution aims to advance the achievement of UN Sustainable Development Goals, notably Clean Water and Sanitation and Responsible Consumption and Production.

## Supporting Information

Additional supporting information can be found online in the Supporting Information section. **Supporting Fig. S1**: SEM micrograph of raw *Luffa cylindrica* prior to polyaniline coating and acid doping.

## Author Contributions

**Rayane Mehennaoui**: conceptualization, methodology, investigation, writing original draft. **Soraya Merzouki**: data curation, formal analysis, writing review and editing. **Salah Neghmouche Nacer**: validation, supervision, resources, project administration. **Abderrahmane Mehennaoui**: software, visualization, formal analysis. **Stefania Garzoli**: supervision, writing review and editing, funding acquisition**. Fadila Louafi**: methodology, resources, investigation.

## Funding

The authors have nothing to report.

## Conflicts of Interest

The authors declare no conflicts of interest.

## Supporting information

Supplementary Material

## Data Availability

The datasets generated and/or analyzed during the current study are available from the corresponding author on reasonable request.
