# Supplementary figures and images for: Impact of Acid Dopants (HCl vs. H2SO4) on the Structure, Performance, and Reusability of Bio‐Based Luffa/Polyaniline Composites for Methylene Blue Adsorption
Source: ChemistryOpen. 2026 May 21;15(6):e70185. doi: 10.1002/open.70185 (PMC13239916; doi:10.1002/open.70185)

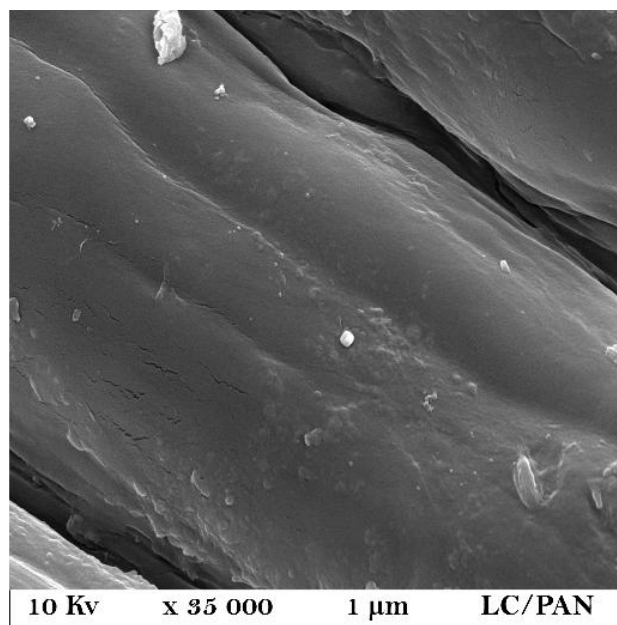

**Figure S1.** SEM micrograph of raw *Luffa cylindrica* prior to polyaniline coating and acid doping.

Supplement: Supplementary file 1 — Supplementary Material [file OPEN-15-e70185-s001.pdf]
